# Amphibian chytridiomycosis: a review with focus on fungus-host interactions

**DOI:** 10.1186/s13567-015-0266-0

**Published:** 2015-11-25

**Authors:** Pascale Van Rooij, An Martel, Freddy Haesebrouck, Frank Pasmans

**Affiliations:** Laboratory of Veterinary Bacteriology and Mycology, Department of Pathology, Bacteriology and Avian Diseases, Faculty of Veterinary Medicine, Ghent University, Merelbeke, Belgium

## Abstract

Amphibian declines and extinctions are emblematic for the current sixth mass extinction event. Infectious drivers of these declines include the recently emerged fungal pathogens *Batrachochytrium dendrobatidis* and *Batrachochytrium salamandrivorans* (Chytridiomycota). The skin disease caused by these fungi is named chytridiomycosis and affects the vital function of amphibian skin. Not all amphibians respond equally to infection and host responses might range from resistant, over tolerant to susceptible. The clinical outcome of infection is highly dependent on the amphibian host, the fungal virulence and environmental determinants. *B. dendrobatidis* infects the skin of a large range of anurans, urodeles and caecilians, whereas to date the host range of *B. salamandrivorans* seems limited to urodeles. So far, the epidemic of *B. dendrobatidis* is mainly limited to Australian, neotropical, South European and West American amphibians, while for *B. salamandrivorans* it is limited to European salamanders. Other striking differences between both fungi include gross pathology and thermal preferences. With this review we aim to provide the reader with a state-of-the art of host-pathogen interactions for both fungi, in which new data pertaining to the interaction of *B. dendrobatidis* and *B. salamandrivorans* with the host’s skin are integrated. Furthermore, we pinpoint areas in which more detailed studies are necessary or which have not received the attention they merit.

**Electronic supplementary material:**

The online version of this article (doi:10.1186/s13567-015-0266-0) contains supplementary material, which is available to authorized users.

## Table of contents

1 Introduction

2 The agents *Batrachochytrium dendrobatidis* and *Batrachochytrium salamandrivorans*

2.1 History and taxonomy

2.2 Morphology, lifecycle and physiology

2.3 Epidemiology

3 The disease chytridiomycosis

3.1 Clinical signs

3.2 Pathology

4 Pathogenesis

4.1 Colonization of amphibian skin

*4.1.1 Interaction with the mucus barrier*

*4.1.2 Adherence to host surfaces*

*4.1.3 Invasion of the epidermis*

4.2 Impairment of the skin function

4.3 Host defenses against chytrid infection

*4.3.1 Innate immune defenses*

*4.3.1.1 Antimicrobial peptides*

*4.3.1.2 Antifungal metabolites*

*4.3.1.3 Lysozyme*

*4.3.2 Acquired immune defenses*

*4.3.3 Immune evasion by chytrid fungi*

4.4 Concepts of susceptibility, tolerance and resistance in chytridiomycosis

4.5 Mediators of chytrid infection dynamics and disease outcome

*4.5.1 Host factors*

*4.5.2 Pathogen virulence*

*4.5.3 Impact of environmental factors*

*4.5.4 Co‑infection with multiple pathogens*

5 Conclusions

Additional files

## Introduction

Amphibians worldwide are dwindling both in numbers and distribution area. According the latest assessment of the IUCN Red List of Threatened Species (update 2015) at least 41% of all extant amphibian species is at risk of extinction [1]. Habitat destruction, alteration and fragmentation, commercial over-exploitation for pet-trade and food, introduction of non-native species, infectious diseases and climate disturbance have been identified as stressors of decline [2].

In the late nineties, sudden mass mortalities in amphibian populations from pristine or protected areas were observed. Especially in biodiversity hotspots like Central America, the Caribbean and Australia, amphibians expirienced “enigmatic” declines. The chytridiomycete fungus, *Batrachochytrium dendrobatidis*, found to parasitize on amphibian skin, was identified as prevailing cause of these amphibian declines (see e.g. [3, 4]). This fungal infection disturbs the vital function of the skin, e.g. respiration and the maintainance of the water balance, and is often lethal [5]. However, the effects of chytridiomycosis on amphibian species and even within one species are variable. While some tolerant amphibians are able to keep infection levels below a lethal threshold and function as carrier species/supershedders of the infective agent, others are susceptible and develop severe lethal disease (see e.g. [6]).

*B. dendrobatidis* is currently present almost everywhere amphibians occur [7]. Until 2010, in Northern Europe it occurred in amphibian populations in a coexistence steady state [8, 9], with only rare reports of *B. dendrobatidis*-linked mortality [10, 11]. Starting from 2010, a steep enigmatic decline was observed in an endangered but stable population of fire salamanders (*Salamandra salamandra*) in the south of the Netherlands [12]. In 2013, only 4% of the population remained. None of the mortalities could be attributed to *B. dendrobatidis* or any other known viral or bacterial amphibian pathogen. About forty individuals were removed from their original habitat and kept ex situ for starting up a breeding programme intended for later reintroduction. Finally, a new chytrid fungus named *Batrachochytrium salamandrivorans* was isolated from the skin lesions of fire salamanders found dead during the Dutch decline event [13]. This novel fungus is currently causing disease outbreaks in, at least, fire salamander populations in the Netherlands [12], Belgium [14] and Germany [15].

Given the huge impact on amphibian populations, chytridiomycosis is sharply phrased as “the worst infectious disease ever recorded among vertebrates in terms of the number of species impacted, and its propensity to drive them to extinction” [2]. Despite this and the plethora of studies focusing on its causative agents, the complex host-pathogen interactions taking place during the act of infecting are far from being understood. With this review we aimed to sketch a general background on host, pathogens and disease and to provide the reader with a comprehensible overview of the complex host-pathogen interactions. At the same time, this opportunity permits to integrate new findings and to identify areas to which future research should be oriented.

## The agents *Batrachochytrium dendrobatidis* and *Batrachochytrium salamandrivorans*

### History and taxonomy

**Fig. 1:**
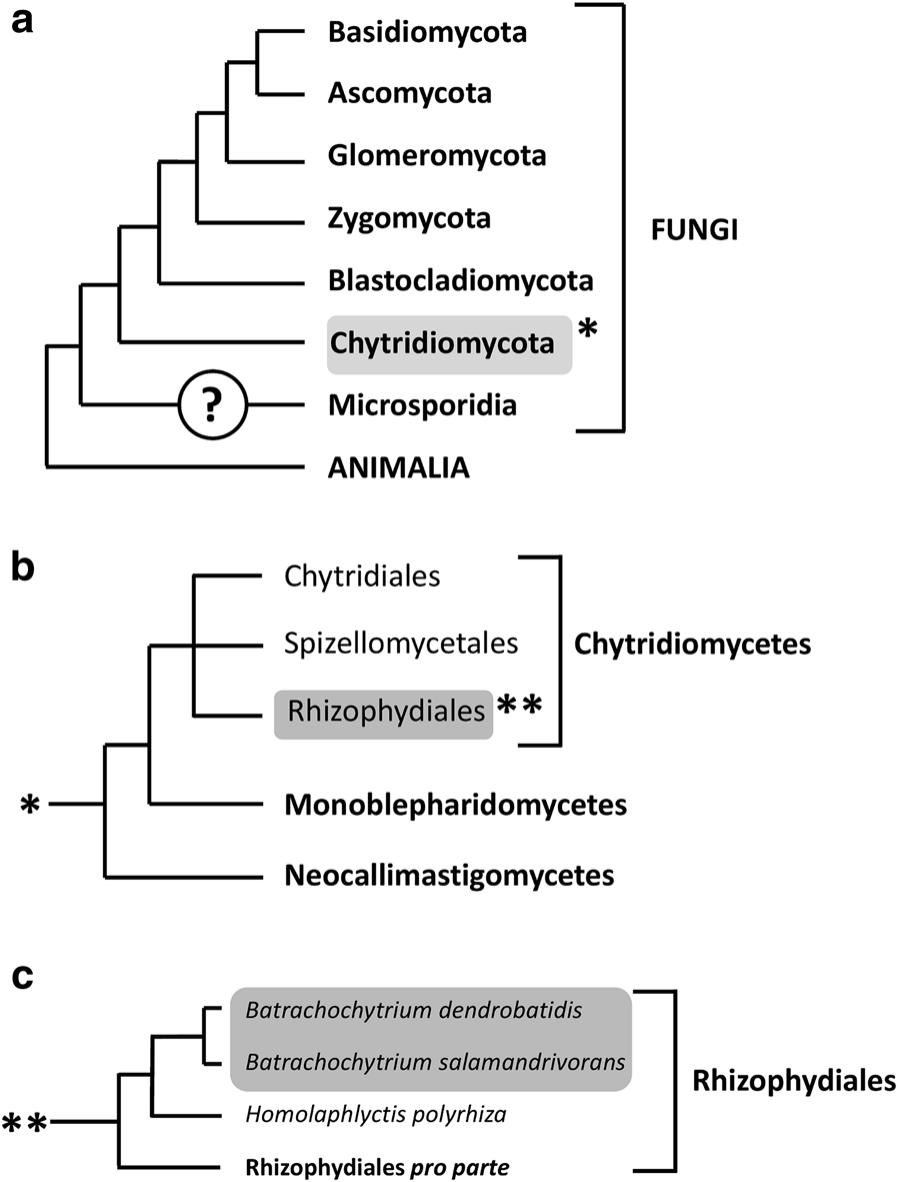
Phylogeny and classification of the genus *Batrachochytrium*. Cladogram showing the taxonomic position of *Batrachochytrium dendrobatidis* and *Batrachochytrium* *salamandrivorans* within the fungal kingdom (**a**), the phylum Chytridiomycota (**b**) and order of the Rhizophydiales (**c**). The position of the Microsporidia remains uncertain. Branch lengths are not proportional to genetic distances. The topology is derived from Martel et al. [13], Longcore et al. [16] and Hibbett et al. [150]

*B. dendrobatidis* and *B. salamandrivorans* belong to the Chytridiomycota, a phylum of “lower fungi” (Fig. 1a). These primitive microscopic fungi have a non-mycelial morphology, characterized by motile flagellated spores or so-called zoospores. Most chytrid fungi inhabit moist soil or fresh water and are in essence saprobic or parasitic on plants, algae or invertebrates [16, 17]. *B. dendrobatidis* and *B. salamandrivorans*, together with *Ichthyochytrium vulgare* (a rare parasite in the skin and gills of freshwater fish) [18] are unique within their phylum in infecting vertebrate hosts.

*B. dendrobatidis* and *B. salamandrivorans* are currently placed in the order of the Rhizophydiales (Figs. 1b and 1c). Their closest relative is the non-pathogenic saprobic *Homolaphlyctis polyrhiza* (Fig. 1c). Inferred from nuclear protein-coding genes, estimates of when *B*. *dendrobatidis* diverged from *B. salamandrivorans* run up to approximately 67.3 million years ago [14].

### Morphology, lifecycle and physiology

**Fig. 2:**
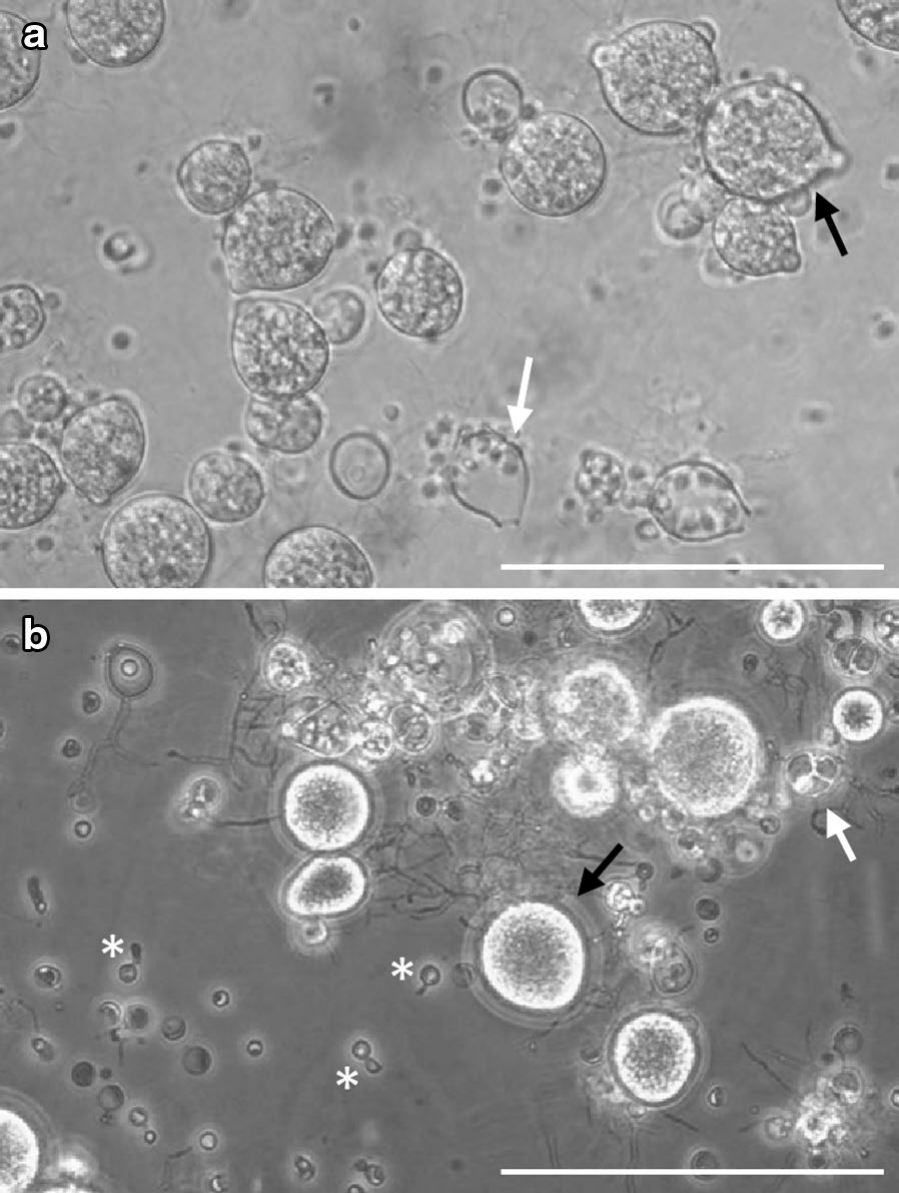
Morphology of *Batrachochytrium* species in culture. **a** Culture of *B. dendrobatidis* on tryptone/gelatin-hydrolysate/lactose (TGhL)-broth, showing abundant mature zoosporangia (black arrow) containing zoospores and empty, discharged sporangia (white arrow); **b** In culture (TGhL-broth) *B. salamandrivorans* is characterized by predominant monocentric thalli (black arrow), few colonial thalli (white arrow) and zoospore cysts with germ tubes (asterisk); scale bars 100 µm

Both *B. dendrobatidis and B. salamandrivorans* have two main life stages as illustrated in Fig. 2: a motile zoospore with a single posteriorly directed flagellum and reproductive body or thallus in which asexual zoospores are produced termed zoosporangium [16]. Sexuality has been scantly documented within the Chytridiomycota [17] and neither *B. dendrobatidis* nor *B. salamandrivorans* have yet been observed in culture to reproduce sexually. However, as several genetic studies have identified *B. dendrobatidis* isolates with a hybrid genotype, sexual recombination and hybridization must have been important mechanisms in its evolutionary history [19–21]. *B. dendrobatidis* is aneuploid, with copy numbers of the chromosomal regions (contigs) within a single isolate running up to 5 [20–22], while data on the ploidy of *B. salamandrivorans* are lacking.

**Fig. 3:**
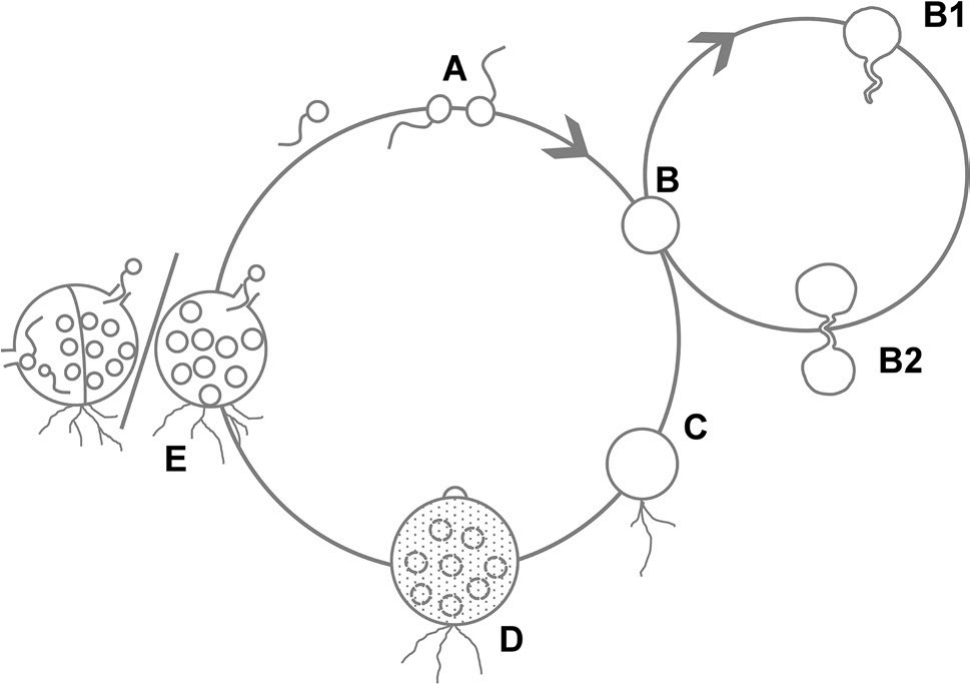
The lifecycle of *Batrachochytrium* species in culture. In culture *Batrachochytrium dendrobatidis* continues the life cycle stages A–E, while in *Batrachochytrium salamandrivorans* additional life cycle stages B1-B2 are observed: (A) flagellated motile zoospores; (B) encysted zoospore; (B1) germling with germtube; (B2) transfer of the cell contents into a newly formed thallus; (C) zoospore cyst with rhizoids; (D) immature sporangium; (E) mature monocentric zoosporangium with discharge tube (at the right), colonial thallus containing several sporangia, each with their own discharge tube (at the left). Modified from Berger et al. [23]

In vitro, *B. dendrobatidis* thrives best in tryptone-gelatin hydrolysate-lactose (TGhL) broth or 1% tryptone broth. The lifecycle of *B. dendrobatidis* in culture, from zoospore to zoosporangium takes 4 to 5 days at 22 °C [23]. *B. salamandrivorans* grows well in TGhl-broth or broth containing peptonized milk, tryptone and glucose (PmTG) and has a lower thermal preference than *B. dendrobatidis*; at 15 °C, its lifecycle in culture is completed within 5 days [12]. Their lifecycle in culture is similar. As shown in Fig. 3, the zoospore first encysts by developing a cell wall and absorbing its flagellum, to finally form a germling with fine tread-like rhizoids. The maturing germling develops into a zoosporangium in which the cytoplasm cleaves mitotically to form new zoospores. Zoosporangia are predominantly monocentric (a thallus containing a single sporangium) and rarely colonial (a thallus containing more than one sporangium). Discharge papillae or tubes, blocked inside by a plug, are formed during the growth of the sporangium. At maturity the plug dissolves and the zoospores are released into the environment to continue their lifecycle [16, 23]. Distinctive features of *B. salamandrivorans* in culture are its lower thermal preference, the presence of tubular extension or germ tubes arising from the encysted zoospores and from which new sporangia arise and the more abundant colonial thalli (shown in Figs. 2b, 3) [12].

The lifecycle of *B. dendrobatidis* in amphibian skin is largely similar to what is observed in culture. Upon colonization of the host epidermis, the zoospores encyst; the flagellum is absorbed and a cell wall is formed [23]. Next, the zoospore cyst germinates and develops a germ tube that invades the host epidermis. At the tip of the germtube a new sporangium arises [24, 25]. Subsequently, the fungus proliferates intracellularly, within the cells of the stratum corneum and the stratum granulosum. Immature sporangia are carried from the deeper skin layers to the skin surface by differentiating epidermal cells. At the time sporangia have developed discharge tubes and contain mature zoospores, they finally occur in stratum corneum where the zoospores are released in the environment [16, 23]. The lifecycle of *B. salamandrivorans* in amphibian skin has not yet been illustrated in great detail, but is assumed to be similar.

Growth and survival of *Batrachochytrium* species are strongly temperature dependent. Optimal growth of *B. dendrobatidis* is observed between 17 and 25 °C and pH 6–7 [26]. At 10 °C or lower, *B. dendrobatidis* grows slowly. At 28 °C or higher *B. dendrobatidis* ceases growth, while its zoospores are killed within 4 h at 37 °C [26, 27]. In addition, desiccation is poorly tolerated [27, 28] and 5% NaCl solutions are lethal [27]. For *B. salamandrivorans*, the optimum temperatures for growth are between 10 and 15 °C. This chytrid fungus can still grow at 5 °C while temperatures of 25 °C and higher are lethal [13, 29].

Under laboratory conditions, *B. dendrobatidis* grows on a variety of keratin containing substrates such as autoclaved snake skin, 1% keratin agar, frog skin agar, feathers and geese paws [16, 26, 28]. However, keratin is not an essential nutrient for this skin attacking fungus: not only *B. dendrobatidis* grows best in tryptone or peptonized milk [16] but also its extracellular proteases fail to degrade keratin in vitro [26]. Besides keratin, *B. dendrobatidis* is also able to grow on the chitinous carapaces of crustaceans [30]. Like its congener, *B. salamandrivorans* is able to attach and grow on keratinous toe squamae of wild geese (Additional file 1). Notwithstanding, its nutritional needs and preferences remain to be studied in detail.

### Epidemiology

*B. dendrobatidis* has a broad host range and infects at least 520 species of anurans (frogs and toads), urodeles (salamanders and newts) (for details see [31, 32]) and caecilians [33]. Many enigmatic declines and local extinctions of amphibian species in Central-America (with losses up to 40% of the local species) [4, 34], North-America [35], Australia [3] and Southern-Europe have been attributed to this fungus [36].

Unlike *B. dendrobatidis*, *B. salamandrivorans* seems restricted to salamanders and newts. Essential information on host range and actual distribution of *B. salamandrivorans* derives from the work of Martel et al. [13, 14]. From the 35 anuran (frogs and toads), urodelan (salamanders and newts) and caecilian species that have been tested under laboratory conditions, none of the anuran or caecilian species became infected. Especially non-Asian salamander species belonging to the family Salamandridae seem highly susceptible for lethal chytridiomycosis, with the majority of the infected animals succumbing within 2–3 weeks after initial exposure. Several recent broad-scaled studies have assessed the extent and impact of *B. salamandrivorans* on amphibian populations worldwide in more than 7000 samples, covering over 170 amphibian species from across 4 continents (Europe, Asia, North-and South America) [14, 37–39]. So far, the distribution of *B. salamandrivorans* seems limited to Western palearctic salamanders, evidenced by disease outbreaks in the Netherlands [12] and Belgium [14] where it reduces populations of (at least) fire salamanders (*Salamandra salamandra*) below 5% of their original population size. At one of the Dutch outbreak sites [40] and in Flanders (Belgium), wild specimens of the Alpine newt (*Ichthyosaura alpestris*) have been found infected with *B. salamandrivorans*, however disease-mediated declines have not yet been recorded for this species. Very recently, *B. salamandrivorans* outbreaks in captive collections of European salamander species (including *Salamandra* and *Speleomantes* spp.) have been reported in the UK [41] and Germany [15].

Where did these fungi originate and how did they spread? So far, the precise origin of *B. dendrobatidis* is unknown. Africa [42], North-America [43] as well as Asia [44, 45] and the Atlantic Forest of Brazil [21, 46] have been suggested as sites of origin. At the moment the Atlantic Forest of Brazil is suggested as cradle of *B. dendrobatidis*. The Brazilian lineage or genotype of *B. dendrobatidis* (*Bd*Bz) has been enzootic in local amphibian assemblages for at least 100 year and has diverged earliest in the phylogenetic history of the pathogen [21, 46]. But also in Asia, *B. dendrobatidis* was present more than 100 years ago. Recently, Fong et al. [45] could trace the presence of *B. dendrobatidis* in Korea back to 1911, by analysis of museum specimens. It is therefore not inconceivable that several lineages of *B. dendrobatidis* have an independent history. *B. salamandrivorans* is thought to have its origins in Asia. This fungus occurs historically at low levels on salamanders of the families Hynobiidae (*Hynobius*, *Onychodactylus*, *Salamandrella* spp.) and Salamandridae (*Cynops*, *Paramesotriton*, *Tylototriton* spp.) throughout at least Japan, Thailand and Vietnam. The oldest evidence was found in a museum specimen of *Cynops ensicauda* (sword-tailed newt) dating back from 1861 [14]. Two competing hypotheses are proposed to explain the origin of any emerging infectious disease, including chytridiomycosis [47]. Such a disease may result from a pathogen that has been present historically at a low to moderate prevalence, but is rendered more pathogenic by changes in host susceptibility, virulence and/or environment; this is known as the endemic pathogen hypothesis (EPH). Alternatively, a pathogen may arrive in a new geographic area, whether or not by human-mediated dispersal, and encounter naïve host populations, as stated by the “novel” or invasive pathogen hypothesis (NPH). There has been much debate about which of these hypotheses explain most satisfactorily the current distribution of *B. dendrobatidis*. The prevailing opinion about this issue is that this pathogen has been endemic in some parts of its range and novel in others [21]. Some species/populations appear less susceptible than others: *B. dendrobatidis* has been present in several amphibian populations before declines occurred [42, 45, 48] and endemic amphibian populations may coexist with *B. dendrobatidis* without any obvious signs or marked declines, e.g. in sub-Saharan Africa, Asia, northern Europe and Brazil [9, 42, 45, 46]. Together with evidence of environmental factors influencing disease outbreak [7], these observations favor the EPH. Several studies have evidenced that when *B. dendrobatidis* enters a region as an invasive species, amphibian populations decline, in some cases to extinction [4, 34]. More recent studies revealed a much larger genetic diversity in *B. dendrobatidis* isolates than previously recognized, indicating a complex evolutionary history of the pathogen [21, 22].

Regardless of *B. dendrobatidis*’s origin, it is clear that the international trade in live amphibians has paved the way to the dispersal of the pathogen between continents [20, 49]. Especially the African clawed frog (*Xenopus laevis*) and North American bullfrog (*Lithobates catesbeianus*) are notorious in this respect. For instance, more than 94% of the amphibian trade in the US is restricted to *L. catesbeianus*, with more than 20 million of specimens traded over an 8 years’ period [50]. *X. laevis* is widely traded for scientific research [42], whereas *L. catesbeianus* is imported at large scale to mainly the US, South-America, China and Europe for consumption [49, 50]. Both species are highly invasive when introduced into new environments [49]. More importantly, *X. laevis* and *L. catesbeianus* are subclinical carriers of *B. dendrobatidis* infection and act as reservoir, transmitting the infection to naïve native amphibians species [42, 51]. In a recent study, Kolby et al. [52] tested water in which *X. laevis* frogs were transported for the presence of *B. dendrobatidis* and found exceptionally high densities ranging from 3390 to 16 887 zoospore equivalent per liter water. It is clear that incorrect disposal of infected wastewater, involves a serious risk of pathogen pollution.

Transmission among hosts is mainly established by the motile waterborne zoospores [16] or through direct contact with infected amphibians (e.g. during mating) [53]. Infected amphibians may shed considerable loads of zoospores into waterbodies, making them potential environmental reservoirs [52, 54]. It is true that, under sterile conditions, *B. dendrobatidis* can survive in water and moist soil for weeks up to several months [55, 56]. However, little is known on the ecology of free-living *B. dendrobatidis* outside its amphibian host [57] and its ability to persist outside its amphibian host in a natural environment, exposed to micro-invertebrates (see further).

Furthermore, *B. dendrobatidis* is able to saprobically grow on e.g. sterile bird feathers, arthropod exoskeletons, keratinous paw scales of waterfowl and to survive in the gastrointestinal tract of crayfish [16, 28, 30, 55, 56]. Both waterfowl and crayfish have been suggested as potential non-amphibian vectors for *B. dendrobatidis* contributing to the dissemination of *B. dendrobatidis* [28, 30]. *B. dendrobatidis* DNA has also been found on wild Panamian lizards and snakes [58], albeit it is not clear if *B. dendrobatidis* can persist on reptile skin under natural conditions.

Also *B. salamandrivorans* was most likely introduced to Europe through amphibian trade. *B. salamandrivorans* was found in imported Asian salamanders. At least five Asian salamander species (*Cynops pyrrhogaster*, *Cynops cyanurus* and *Paramesotriton deloustali* and *Salamandrella keyserlingii*) could be suitable reservoirs of the disease and are able to shed zoospores for at least 5 months without necessarily developing clinical disease [14]. This is quite worrysome since *B. salamandrivorans* is highly contagious for susceptible salamander species. Cohousing experiments learn that a contact period of 8 h is sufficient for transmission of *B. salamandrivorans* from infected *Cynops pyrrogaster* to naïve individuals and other species [14]. Moreover, especially fire-bellied newts of the genus *Cynops* are traded in large numbers, exceeding 2 million in an 8-year period [50]. The risk for indroduction of *B. salamandrivorans* in other regions than northern-Europe is thus quite realistic. The study of the survival of *B. salamandrivorans* outside its host and its spread in the environment is still a virgin field. Future research should also focus on possible carriers and non-amphibian vectors favoring this pathogen’s spread.

## The disease chytridiomycosis

### Clinical signs

**Fig. 4:**
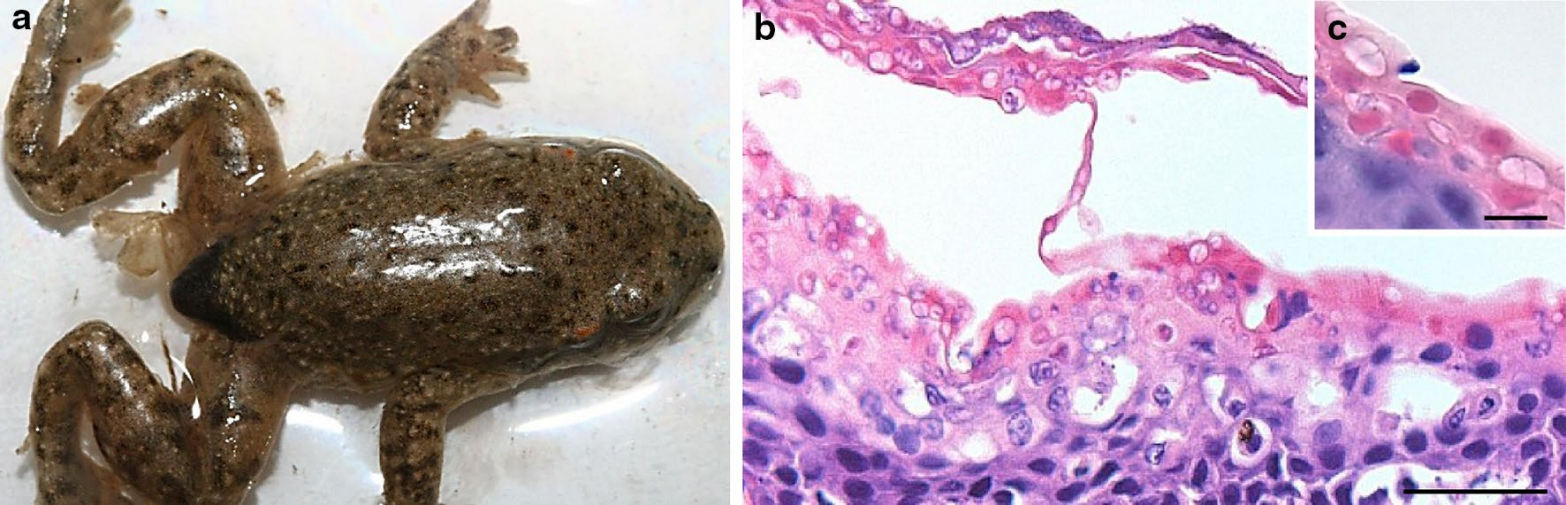
Clinical signs and pathology associated with infection due to *Batrachochytrium dendrobatidis.* **a** Naturally infected moribund common midwife toad (*Alytes obstetricans*) showing abnormal posture (abduction hind legs) and loose sloughed skin; **b** section through the ventral skin (drink patch) of the same infected toad; infection is characterized by diffuse epidermal hyperkeratosis and hyperplasia combined with the presence of numerous zoosporangia at various stages of maturation; HE; scale bar 50 µm; **c** detail of intracellular septate zoosporangia; HE; scale bar 10 µm

In anuran larvae, clinical signs of chytridiomycosis due to *B. dendrobatidis* are generally limited to depigmentation of the mouthparts, without morbidity and mortality [3, 59]. However, *B. dendrobatidis* may cause sub-lethal effects, including lethargy or poor swimming abilities, leading to low foraging efficiencies which is reflected in reduction in body size [60]. In metamorphosed amphibians clinical signs are variable and range from sudden death without obvious disease to significant skin disorder but infection may nonetheless elapse asymptomatically. Most common signs of chytridiomycosis are excessive shedding of the skin (Fig. 4a), erythema (redness) or discoloration of the skin [61]. Although uncommon in other species, skin ulcerations occurred in severely infected green tree frogs (*Litoria caerulea*) [62]. In frogs and toads, the skin of the ventral abdomen, especially the pelvic patch (a highly vascularized skin area on the ventral side of the body), feet and toes are predilection sites of infection [62, 63], while in salamanders the pelvic region, fore and hind limbs and the ventral side of the tail seem more prone to infection [64]. Other clinical signs include lethargy, anorexia, abnormal posture (abduction of the hind legs) (Fig. 4a), neurological signs such as loss of righting reflex and flight response [61]. In bolitoglossine salamanders, chytridiomycosis has been associated with tail autotomy (self-induced separation of the tail from the body) [65].

**Fig. 5:**
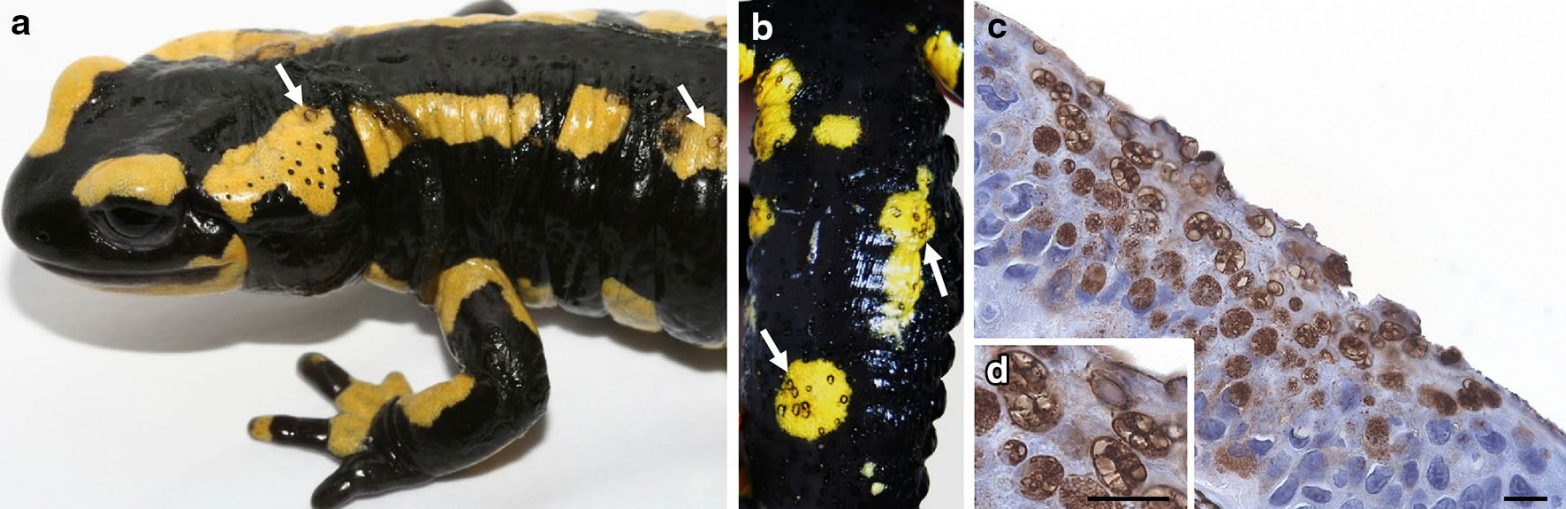
Clinical signs and pathology associated with infection due to *Batrachochytrium salamandrivorans*. **a** a naturally infected fire salamander (*Salamandra salamandra*) found during a *B. salamandrivorans*-outbreak (Robertville, Belgium) showing several ulcers (white arrows) and excessive skin shedding; **b** extensive ulceration (white arrows) at the ventral side of an infected fire salamander; **c** skin section through an ulcer evidences abundant intracellular colonial thalli in all epidermal skin layers; immunohistochemical stain with polyclonal antibodies to *B. dendrobatidis*; scale bar 10 µm; **d** magnification of the intracellular colonial thalli from micrograph **c**; immunohistochemical stain; scale bar 10 µm

Chytridiomycosis due to *B. salamandrivorans* in metamorphosed urodelans is most strikingly characterized by multifocal superficial erosions and extensive epidermal ulcerations all over the body, as shown in Figs 5a, b. Coinciding clinical signs include excessive shedding of the skin, anorexia, apathy, ataxia and death [13]. In contrast, the pathogen appears harmless to, at least, urodelan larvae of *S. salamandra* as experimental inoculation did not result in colonization of the larvae (Additional file 2).

### Pathology

In metamorphosed amphibians, chytridiomycosis due to *B. dendrobatidis* is diagnosed by the presence of immature chytrid thalli or maturing sporangia found intracellularly in the keratinized layers of the skin as shown in Figs 4b, c. Infection is mainly associated with a mild to severe irregular thickening (hyperkeratosis) of the outermost keratinized layers of the epidermis (the stratum corneum and stratum granulosum), erosion of the stratum corneum, and increased tissue growth (hyperplasia) of the stratum spinosum which lies beneath the keratinized superficial skin layers. Also ulceration of the skin may occur [62]. Other pathological changes in the epidermis adjacent to the foci of infection include mild focal necrosis, intercellular edema (spongiosis), cytoplasmic degeneration with minimal to mild inflammation and vacuolation of the deeper cell layers [23]. Dissemination to the deeper layers of the skin or the internal organs does not occur [61]. However, in severely infected *Litoria caerulea* an atypical pathology including severe congestion of the skin and internal organs may be observed [62]. In anuran larvae, infection is limited to colonization of the keratinized mouthparts and is absent in the epithelia of body, limbs, tail, mouth and gills. Infection is accompanied with minimal pathology, predominantly consisting of mild hyperkeratosis [3, 66]. Apart from rare exceptions (e.g. in *Ambystoma* spp.) data about infection in urodelan larvae are lacking. It is noteworthy that in rule urodelan larvae are carnivorous and have true teeth instead of keratinous mouthparts.

In contrast, the pathology corresponding with *B. salamandrivorans* infection in salamanders is quite straightforward. The characteristic erosive skin lesions are associated with the presence of numerous intracellular colonial thalli that spread all over the epidermis and marked necrosis of the adjacent keratinocytes (Figs 5c, d). Hyperplasia and hyperkeratosis, the hallmarks of *B. dendrobatidis* infection, are absent [13].

## Pathogenesis

With the availability of *B. dendrobatidis*’s full genome, genome based studies have led to an improved understanding of host-pathogen dynamics and the identification of several putative pathogenicity factors with high specificity for skin-related substrates, facilitating colonization or causing host damage. Nevertheless, processes taking place during the whole infection process at molecular and cellular level such as cell signaling, induction of cytoskeletal change and so on, are still less well or barely understood and definitely merit more attention. As research on the recently emerged *B. salamandrivorans* was launched only 2 years ago, our current knowledge is still at its infancy. For now, we are still groping in the dark about, for example, what factors make *B. salamandrivorans* specific to salamanders and newts, how infection is established and ultimately leads to mortality, and whether or not effective immune responses are elicited during infection. Identification of pathogenicity factors in *B. salamandrivorans*, involved in its pronounced clinical manifestation and its rapid disease progression, is still long coming. The genome of *B. salamandrivorans* is not yet fully sequenced but whenever available, comparative studies of both fungi’s expression profiles may considerably accelerate our insight in factors underlying their differential disease dynamics.

### Colonization of amphibian skin

The different steps in the infection process by *B. dendrobatidis* comprise attraction to a suitable host, attachment of zoospores to the host skin, zoospore germination, germ tube development and penetration into the skin cells, followed by invasive growth in the host skin, finally resulting in the loss of the host cell cytoplasm. In this chapter, each of these crucial steps will be discussed in detail.

#### Interaction with the mucus barrier

**Fig. 6:**
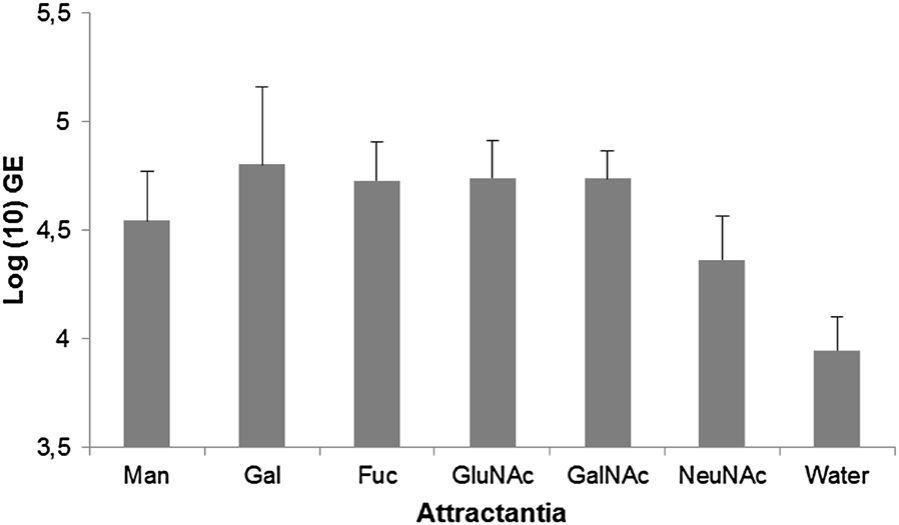
Chemotaxis of *Batrachochytrium dendrobatidis* toward free integumental sugars. The sugars α-d-mannose (Man), α-d-galactose (Gal), α-l-fucose (Fuc), β-d-*N*-acetylglucosamine (GluNAc), α-d-*N*-acetylgalactosamine (GalNAc), *N*-acetylneuraminic acid (NeuNAc) or sialic acid were tested as attractans at a 0.1 M concentration, using a traditional capillary tube test. Water was used as vehicle and controle attractans. Genomic equivalents (GE) of *B. dendrobatidis* zoospores in the capillaries were quantified after a 90 min using quantitative real-time PCR. Mean ± standard error of three independent experiments are presented

**Fig. 7:**
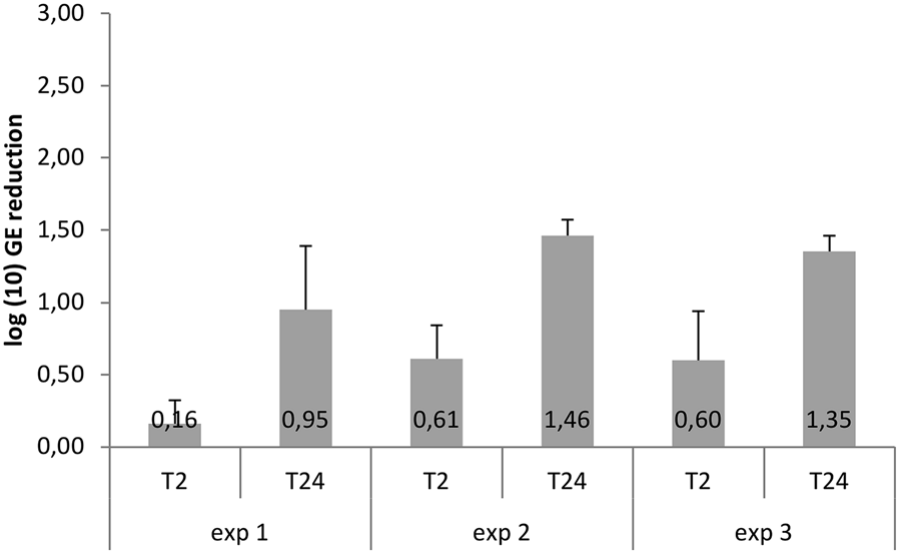
Zoosporicidal activity of *Xenopus laevis* skin mucus at physiological concentrations. Killing activity is expressed as log(10) viable spores added to the skin secretions—log(10) viable spores recovered after 2 and 24 h incubation. Results are presented as mean genomic equivalents of *B. dendrobatidis* ±standard error (SEM). Sample size (n) for time point (T) = 3

Directed movement or chemotaxis of flagellated pathogens towards a suitable host and nutrient substrate are often crucial in the establishment of colonization (for review see [67]). There is proof of *B. dendrobatidis* responding positively to certain cues from host or vector origin. Chemotaxis to keratinous toe scales of geese [28] as well as to commercially available keratin and its main constituent amino acid, cysteine [68] has been reported. As numerous bacterial pathogens are found to be attracted to mucus, with some pathogens able to metabolize components of mucus [67], the question arises whether *B. dendrobatidis* could also migrate actively towards the mucous layer covering the amphibian epidermis or one of its components. In search of a suitable amphibian host, *B. dendrobatidis* zoospores indeed will first come into contact with skin mucus. The main component of mucus are mucins or mucin glycoproteins. In the African clawed-frog (*X. laevis*) the carbohydrate portion of mucins includes the sugars α-l-fucose, α-d-*N*-acetylgalactosamine, β-d-*N*-acetylglucosamine, *N*-acetylneuraminic acid or sialic acid, α-d-galactose and α-d-mannose. This spectrum of sugars, constituting the so-called integumental free sugars, was also found in its upper epidermis, and in the epidermises of the caecilian *Ichtyophis kohtaoensis*, the smooth newt (*Lissotriton vulgaris*) and the edible frog (*Pelophylax* kl. *esculentus*) [69]. Indeed, using a disc method, positive migration of *B. dendrobatidis* towards skin mucus isolated from *X. laevis* was observed (Table 1; also see Additional file 3). Using a capillary tube chemotaxis assay combined with real-time PCR quantification we found that the free sugars in mucus and amphibian skin are chemotactic (Fig. 6; also see Additional file 4). The odds of *B. dendrobatidis* zoospores being attracted to sugar were approximately 3–9 times higher compared to water (Table 2). Amphibian skin mucus not only offers protection against abrasive damage and dehydration, but is also thought to serve as a critical barrier against colonization by pathogens. Mucus contains several interdependent host factors including antimicrobial peptides (AMPs), lysozymes and mucosal antibodies as well as microbial-community factors, including symbiotic skin bacteria producing antifungal metabolites (for review see [70]), which will be discussed in a later part of this review (Sect. 4.3). This micro-ecosystem of the mucus is referred to as the mucosome. Before zoospores can establish a successful colonization of the host skin, they must first resist the defense factors of the mucosome. The mucosome in its entirety may reduce the infection load on the skin during the first 24 h that are critical for colonization and establishing skin infection [24]. Indeed, in vitro exposure of *B. dendrobatidis* zoospores to the skin mucus, covering the epidermis of *X. laevis*, with all residing defenses in physiological concentrations, causes up to a 3 to 20-fold reduction in the amount of viable zoospores within 2–24 h after exposure (Fig. 7; also see Additional file 5). All in all, these data suggest that skin mucus plays a dual role in pathogenesis: although mucus is attractive for zoospores, it constitutes a defensive barrier, limiting invasion to the underlying epithelium by trapping and reducing the amount of infective zoospores on the skin.

**Table 1:** Chemotaxis of *B. dendrobatidis* zoospores towards mucus and water

Time (min)	Attractans			
	Mucus		Water	
	Mean zoospores ± SEM	% Motile/immobile	Mean zoospores ± SEM	% Motile/immobile
0	0	0	0	0
45	76.8 ± 64.2	38.1	0	0
90	152.5 ± 112.1	29.0	0.75 ± 1.5	25.0

Mean numbers of zoospores with standard errors (mean ± SEM) and the percentage (%) of motile zoospores observed in 4 assays, is given for each time point

**Table 2:** Predictors of chemotaxis

	OR (A/NA)	95% CI	*P* value
Man	4.93	1.77–5.05	<0.0001
Gal	9.14	8.93–9.30	<0.0001
Fuc	6.95	6.82–9.96	<0.0001
GluNAc	6.59	6.45–6.74	<0.0001
GalNAc	4.49	4.39–4.57	<0.0001
NeuNAc	3.02	2.94–3.09	<0.0001

For each of the sugars tested, the odds of zoospores being attracted (OR) based on the group ratio attracted (A) versus non-attracted (NA), is given with the corresponding 95% confidence interval (CI) and *P* value

*Man* mannose, *Gal* galactose, *Fuc* fucose, *GluNAc* *N*-acetylglucosamine, *GalNAc* *N*-acetylgalactosamine, *NeuNAc* *N*-acetylneuraminic acid

#### Adherence to host surfaces

**Fig. 8:**
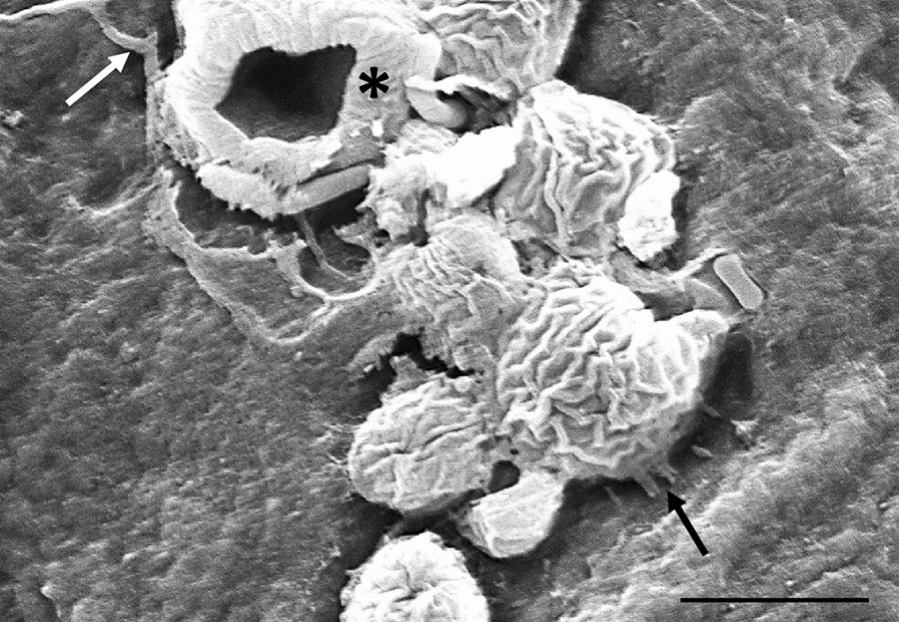
Adhesion of *Batrachochytrium dendrobatidis* to *Xenopus laevis* skin. Adhesion to the epidermal surface is established both by tubular projections, possibly adhesins (black arrow) and rhizoids (white arrow). Some encysted zoospores have collapsed (asterisk) due to cell hollowing; scale bar 5 µm

So far, the mechanisms and kinetics of adhesion of *B. dendrobatidis* to amphibian skin have only received limited attention. Adhesion has been documented in explanted amphibian skin and occurs within 2–4 h after exposure to zoospores [24]. Zoospores mature into thick-walled cysts on the host epidermis and often cluster in foci of infection. Cysts are anchored to the skin surface by fine fibrillar projections as shown in Fig. 8 (also see Additional file 6) that resemble the fibrillar adhesins documented for pathogenic fungi affecting human skin e.g. *Trichophyton mentagrophytes* (reviewed in [71]). However, the composition of these fibrils remains to be defined. Several genes encoding proteins involved in cell adhesion such as vinculin, fibronectin and fasciclin have been identified in the *B. dendrobatidis* genome, and are brought more to expression in sporangia than in zoospores [72]. When grown on pulverized host tissue, at least 11 potential adhesion genes which are almost all specific to *B. dendrobatidis*, show an increased expression [73] and require further characterization. The gene expression of *B. dendrobatidis* has not yet been mapped during its early interactions with amphibian skin (neither in vivo nor in vitro) and as such the exact factors mediating adhesion remain uncertain. Adherence mechanisms may include the action of mainly agglutinin-like and lectin-like proteins as described for numerous pathogens. Further research should focus on the identification of adhesins and their respective receptors on the host surface, especially since these insights could open new perspectives for prevention and treatment of chytrid infection.

*B. dendrobatidis* is equipped with a chitin binding module (CBM18) that is hypothesized to facilitate survival on its amphibian host. Chitin, a polymer of *N*-acetylglucosamine is the main component of the fungal cell wall. CBM’s found in other pathogenic fungi function as competitor of, and limit access for foreign chitinases by binding to the chitin of their proper cell wall. In analogy, a key role of CBM18 in the pathogenesis and protection against host-derived chitinases is suggested. In addition, CBM18 would also allow attachment of the pathogen to non-host chitinous structures (e.g. insect or crustacean exoskeletons) allowing vectored disease spread [30, 74].

#### Invasion of the epidermis

**Fig. 9:**
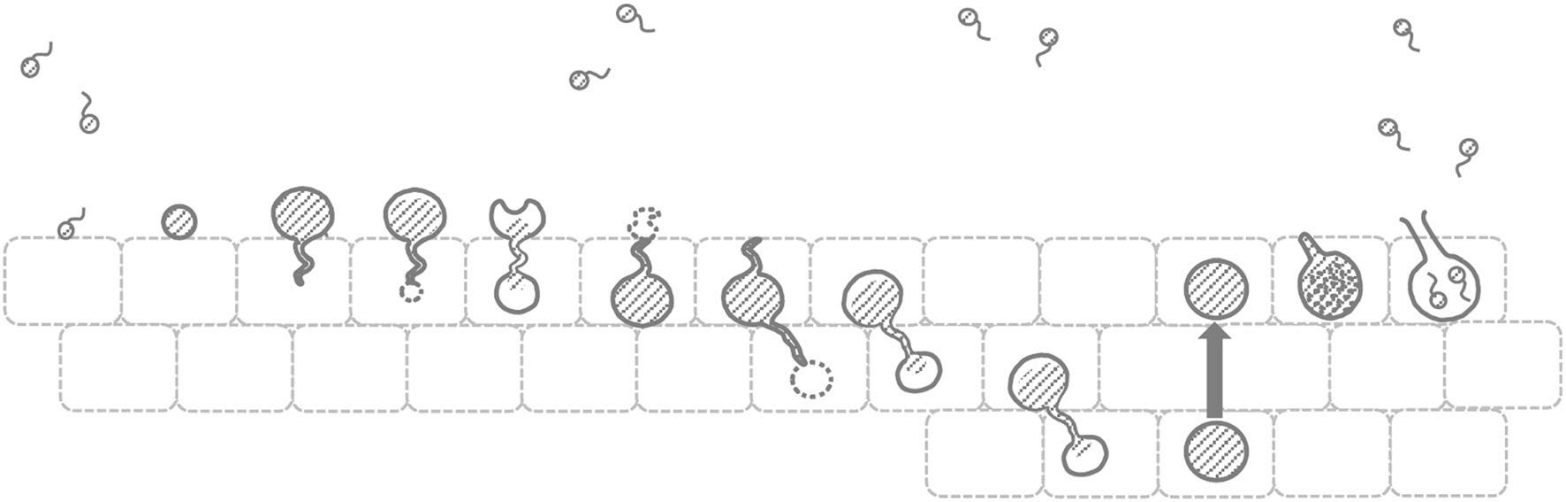
Infection cycle of *Batrachochytrium dendrobatidis* in a susceptible host. The endobiotic lifecycle includes successively germ tube mediated invasion, establishment of intracellular thalli, spread to the deeper skin layers, upward migration by the differentiating epidermal cell to finally release zoospores at the skin surface

**Fig. 10:**
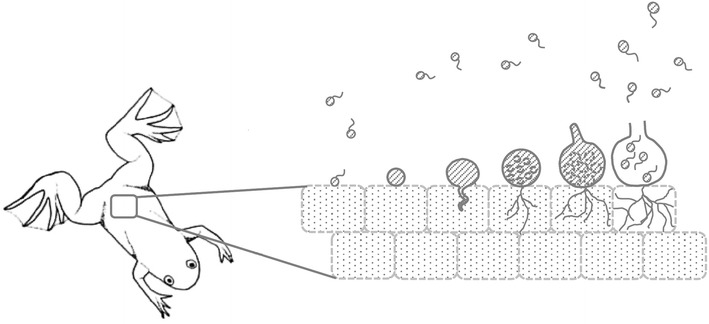
Epibiotic lifecycle of *Batrachochytrium dendrobatidis*. The epibiotic lifecycle observed in skin explants of *Xenopus laevis* includes germ tube mediated invasion, outgrowth of a rhizoidal network, uptake of host cell cytoplasm as nutrient for the growing and maturing chytrid thallus upon the skin surface

The mechanism of host cell entry, intracellular development and spread within the skin has been documented in a skin explant model [24, 75] as well as for experimentally infected frogs [25]. In general, *B. dendrobatidis* develops endobiotically, i.e. with sporangia located inside the host cell and is achieved within 24 h after initial exposure. Colonization is established via a tubular extension or germ tube arising from the zoospore cyst that penetrates the host cell membrane and enables transfer of genetic material into the host cell. Then, the distal end of the germ tube swells and gives rise to a new intracellular chytrid thallus. The pathogen then uses the same tactics to spread to deeper skin layers: older “mother” thalli develop rhizoid-like structures spreading to deeper skin layers, form a swelling inside the host cell to finally give rise to a new “daughter” thallus. Figure 9 presents the putative lifecycle of *B. dendrobatidis* in the skin of susceptible amphibian species, resulting from compilation of all available data [23–25]. The presence of intracellular chytrid thalli clearly contributes to the disease progression in susceptible animals, but the question remains whether “internalization” of the chytrid fungus aids to evade the innate host defences. As such, more research is needed to define the biological advantage of spreading chytrid propagules to deeper skin layers. Conversely, in explanted skin of the infection tolerant *X. laevis* the pathogen develops merely epibiotically, i.e. with sporangia developing upon the skin (shown in Fig. 10). Here, the affected epidermal cells seem to be solely used as nutrient source for the growing sporangium upon the epidermis [24]. Due to the lack of conclusive histological evidence, it is not clear how infections manifest in this species under natural conditions. As this “saprobic” type of development has only been observed in vitro, more observations are necessary.

*B. dendrobatidis* only colonizes keratinized, stratified epidermis. In anuran larvae, colonization is limited to the keratinized mouthparts, i.e. tooth rows and jaw sheats, and is absent in the epithelia fated to keratinize at metamorphosis, i.e. body, limbs, tail, mouth and gills [3, 66]. Studies in *Mixophyes fasciolatus* and *Osteopilus septentrionalis* larvae learn that during metamorphosis colonization of the skin by *B. dendrobatidis* progresses following the distribution of keratin. Shortly before metamorphosis (approx. Gosner stage 38) the epithelia on the feet begin to stratify and keratinize. Then, at metamorphosis (approx. Gosner stage 40), keratin degrades from the mouthparts before the epithelia on the rest of the froglet’s body begin to keratinize. At that time *B. dendrobatidis* infection transitions from the mouthparts to the hindlimbs [66, 76].

Our knowledge about the interactions between *B. salamandrivorans* and its urodelan host is only a minute snippet. The limited number of publications concerning this novel pathogen evidence a rapid disease development in infected urodelans [13, 14]. Skin invasion correlates with host susceptibility. Inoculation of healthy susceptible salamanders is followed by invasion and intracellular colonization of the skin within 24 h and can cause mortality within 2 weeks [14]. As *B. salamandrivorans* develops germ tubes in culture, invasion and spread in the host epidermis is most likely also germ tube mediated, following the same pattern as *B. dendrobatidis*, but certainly merits further dissection. Due to its particular host specificity, the mechanisms of attraction to the host, host recognition and adherence require further study.

### Impairment of the skin function

The skin of amphibians is of vital importance for their survival. Not only it functions as a sensory organ, but also plays an important role in osmoregulation, i.e. the regulation of the osmotic pressure of an amphibians’ body fluid, defense, thermoregulation, sex recognition and reproduction. Although most adult amphibians possess lungs, an important part of O_2_/CO_2_ exchange takes place through the skin [77].

We have a quite clear picture of the physical and physiological changes resulting from infection by *B. dendrobatidis* and leading to (lethal) disease, while for its congener, *B. salamandrivorans*, the question remains hitherto unanswered. Severe chytridiomycosis interferes with the skin’s vital functions. Impairment starts with the physical disruption of the epidermis. In human pathogenic fungi causing skin infections, extracellular proteases e.g. serine-, aspartyl- and metallo-proteases play an important role in the invasion of the host skin [71]. These digestive enzymes not only cause damage to host tissue but also impair host defenses. *B. dendrobatidis* possesses a large number of protease encoding genes, that are lacking or far less prominent in its non-pathogenic congeners [21, 78]. In this fungus’ genome two gene families were found, encoding a serine-type protease and a fungalysin metallopeptidase, two candidates aiding in host cell invasion and dissolution of cellular cytoplasma. Indeed, in the laboratory, *B. dendrobatidis* secretes proteases capable of degrading casein, gelatin (a hydrolysed form of collagen) [26, 79] and elastin [79]. Additionally, Brutyn et al. [80] discovered that its zoospores secrete a complex mixture of virulence associated proteins including proteases, biofilm-associated proteins and lipases, compromising skin integrity by disturbing the hosts intracellular junctions. Furthermore, infection due to *B. dendrobatidis* triggers a decreased expression of host genes encoding for essential skin integrity components such as keratin, collagen, elastin and fibrinogen [73].

Physical disruption of the epidermis directly affects the osmoregulatory function of the skin: it impairs the electrolyte transport across the skin, accompagnied by a reduction in transepithelial resistance and leakage of ions, giving rise to ion imbalances, and a reduced ability of frogs to osmoregulate or rehydrate. In fact, in blood samples taken from amphibians with clinical chytridiomycosis significantly reduced plasma sodium, potassium and chloride ion concentrations as well as reduced overall blood plasma osmolality have been observed. Especially, low plasma potassium concentrations (or hypokalemia) that are linked to abnormal cardiac electrical activity and cardiac arrest, are thought to be the proximate cause of death in diseased amphibians (for review see [5]). Many fungal and bacterial pathogens are known to alter both structure and function of the host epidermis and induce changes in water and electrolyte transport by activation or inhibition of ion channels and transporters. Electrolyte transport across the amphibian epidermis is partially accomplished by epithelial sodium channels (ENaC) and sodium/potassium pumps. A study by Campbell et al. [5] shows that chytrid infection is likely to inhibit ENaC, leading to a severely reduced sodium absorption through the skin. Whether a toxin secreted by *B. dendrobatidis* itself or changes in enzyme function or protein expression induced by the fungus cause disruption of normal skin functioning, requires further research.

### Host defenses against chytrid infection

Both innate and acquired immune components contribute importantly to the antimicrobial function of the mucus, as already pinpointed earlier, and will be elaborated in this chapter.

#### Innate immune defenses

##### Antimicrobial peptides

A first innate immune defense mechanism involves the production of AMPs in dermal granular glands. AMPs are small (12–50 amino acid residues), cationic and hydrophobic peptides that can reorganize into an amphipathic (with both a hydrophobic and hydrophilic portion within the same molecule) α-helix when bound to charged residues on target cell membranes. Resulting peptide complexes interact with and penetrate into the cell membrane. The mechanisms of AMP action are under debate and both membrane disruption and cell internalization followed by disruption of intracellular targets have been proposed [81]. Most of our current knowledge concerning amphibian AMPs stems from studies on Anura. Although a vast number of well-studied species lack conventional AMPs (for review see [82]), there is a plethora of data underscoring the effectiveness of AMPs in skin secretions as first line defense against *B. dendrobatidis*, reducing the infection load on amphibian skin to tolerable levels or even clearing them from infection (e.g. [83, 84]). It is not known to which degree anuran AMPs are effective against *B. salamandrivorans*. To date approximately forty anuran AMPs inhibiting *B. dendrobatidis* have been characterized [84]. Both purified and natural mixtures of these AMP’s effectively inhibit in vitro growth of both *B. dendrobatidis* zoospores and sporangia [83–85]. As the infective spores of *B. dendrobatidis* lack a cell wall, disruption of the cellular membrane integrity has been hypothesized [70]. However, it is not clear to which extent these peptides provide protection against chytridiomycosis in vivo. Species with peptides active in vitro such as the mountain yellow-legged frog (*Rana muscosa*) may turn out to be very susceptible for infection in nature [86]. Moreover, the efficacy of skin peptide defenses may vary at species and population level [83, 87]. Also little is known about the activity of AMPs once they are secreted upon the skin. Degradation dynamics of skin peptide defenses in species of the *Pelophylax* complex and in the Northern leopard frog (*L. pipiens*) suggest that once peptides are secreted upon the skin they stay active up to 1–2 h, but are then degraded by host proteases [88, 89]. Unlike for anurans, information about the AMP arsenal in skin secretions of urodelans is scant. In several salamanders species, the antimicrobial action of skin secretions has been attributed to antimicrobial compounds, most probably including AMPs [90, 91]. However, to date only a single antimicrobial peptide (the defensin CFBD) has been described from *Cynops fudingensis* (Fuding fire belly newt) [92], leaving a wealth of novel AMPs to be discovered. To our knowledge, the antimicrobial action of CFBD against both *Batrachochytrium* species has not (yet) been evaluated. Although published data are virtually lacking, AMPs may be involved in the anti-*B. dendrobatidis* activity of salamander skin secretions [90, 91] and potentially play a role in defense against *B. salamandrivorans*.

##### Antifungal metabolites

A second innate immune defense against *B dendrobatidis* infections is provided by secondary metabolites secreted by symbiotic bacteria present on amphibian skin. So far, only 3 inhibitory metabolites secreted by the skin bacterial species *Janthinobacterium lividum*, *Lysobacter gummosus* and *Pseudomonas fluorescens* have been identified, i.e. 2,4-diacetylphloroglucinol (2,4-DAPG), indol-3-carboxaldehyde (I3C) and violacein [93]. These antifungal metabolites aid to maintain infection loads below a lethal threshold and exhibit a dual action. First, these metabolites can inhibit growth of *B. dendrobatidis* both in vitro and in vivo [93–95]. However, it is unknown to what extent these metabolites can inhibit *B. salamandrivorans*. Besides, co-culture of skin bacterial isolates can ultimately lead to secretion of new, more potent metabolites then when grown in monoculture. As such, the inhibitory metabolite tryptophol was found to emerge from co-culturing an unknown *Bacillus* skin bacterium and *Chitinophaga arvensicola* [96]. Similarly, Myers et al. [94] discovered that these metabolites work synergistically with AMPs to inhibit growth of *B. dendrobatidis*, at lowered minimal inhibitory concentrations (MIC) necessary for inhibition by either metabolites or AMP’s. In addition, the metabolites 2,4-DAPG and I3C seem to exert a repellent action on *B. dendrobatidis* zoospores [97]. As for AMPs, variation in pathogen susceptibility among populations is thought to result in part from differences in bacterial skin communities. By comparing bacterial communities on the skin of a declining *Rana muscosa* population and a population coexisting with *B. dendrobatidis*, researchers found a significantly higher number of individuals with culturable bacterial species displaying antifungal properties in coexisting populations than in those at decline. Alteration of this microbial community composition, for example by environmental factors, can considerably increase susceptibility to disease [95]. Conversely, the team of Harris [98] found that addition of the beneficial skin bacterium *J. lividum* to the skin of susceptible *R. muscosa* frogs before experimental exposure to *B. dendrobatidis*, considerably alleviated symptoms of chytridiomycosis and prevented morbidity and mortality. However, mitigation of chytridiomycosis using probiotics will prove challenging as not all symbiotic skin bacteria exhibit broad-spectrum inhibition across isolates of a hypervirulent, globally spread *B. dendrobatidis* lineage (*Bd*GPL) [19, 99].

##### Lysozyme

Another compound with fungicidal potential in amphibian skin mucus is lysozyme [70], but has hitherto not been studied in detail. The natural substrate of lysozyme or murimidase is peptidoglycan, a major component of the bacterial cell wall and polymer of *N*-acetylglucosamine (GlcNAc) and *N*-acetylmuramic acid units. By splitting the β-1,4 bonds lysozyme causes cell lysis. As the fungal cell wall consists mainly of chitin, a polymer of β-1,4 linked GlcNAc units, it is also a potential target for lysozyme. Although we detected lysozyme or lysozyme-like proteins in mucus from *X. laevis*, preliminary MIC assays using commercial lysozyme from chicken egg white (1–128 U/mL), failed to demonstrate any fungicidal effect against *B. dendrobatidis* (Additional file 7). More studies in this domain are necessary to draw further conclusions.

#### Acquired immune defenses

Unlike the innate immune system, the acquired or adaptive immune system provides highly specific protection against pathogens. This immunity strategy involves both cell-mediated and antibody responses. However, what has puzzled many researchers is the apparent absence of a robust immune response in susceptible amphibian species. Mapping of transcriptomic changes in immunological important tissues (skin, liver, spleen, small intestine) from frogs diseased with *B. dendrobatidis*, evidence a decreased expression of immunity-related genes (associated with e.g. lymphocytes, Toll-like receptors, complement pathways) [100, 101] or even a lack of protective response by the adaptive immune system [102]. Besides, there is conflicting information about whether or not a protective immune response can be elicited in amphibians. So far, attempts to immunize frogs by subcutaneous or intraperitoneal injection of formalin (*Rana muscosa*, see [103]) or heat-killed *B. dendrobatidis* (*Bufo boreas*, see [70]) failed to elicit an acquired immune response against *B. dendrobatidis*. Only in *X. laevis*, *B. dendrobatidis*-specific IgM, IgX (mammalian IgA-like) and IgY (mammalian IgG-like) antibodies were found in skin mucus after injection with heat-killed zoospores [85]. Usually, antibodies play an important role in neutralizing pathogens or presenting them to other components of the immune system for destruction [70]. In vitro, the mucosal antibodies elicited in *X. laevis* frogs indeed bind with *B. dendrobatidis* zoospores and are suggested to limit colonization of the skin to mild and non-lethal infections [85], but their contribution to real-time protection remains to be determined. As *B. dendrobatidis* infections naturally occur in the skin, it seems likely that introduction of *B. dendrobatidis* antigens directly into the skin and targeting immune effectors in the skin may be more effective [70]. However, evidence for effectiveness of vaccination in form of prior infection by topical exposure of the skin to live or heat-killed *B. dendrobatidis* is mixed. Several studies have reported a higher survival, reduction in the infection load or complete clearance in frogs that have been repeatedly exposed to *B. dendrobatidis* than in immunologically naïve frogs [85, 104], while in others pre-exposure had no such effect [105]. To our knowledge, immunization of salamanders against *B. salamandrivorans* has not yet been attempted.

#### Immune evasion by chytrid fungi

Evasion of host immune recognition and inhibition of antifungal defenses are commonly seen in fungal pathogens. Also for *B. dendrobatidis* there is ample proof of active suppression of host responses coming from genetic, peptidomic, in vitro and in vivo-immune studies [100–102, 106–109].

Ellison et al. [101] found that in highly susceptible harlequin frogs (*Atelopus zeteki*) *B. dendrobatidis*-specific immune responses are indeed elicited, but are not effective. Ineffective immune pathway activation and antibody production have been suggested as underlying mechanisms [101]. A breakthrough in research was the discovery that *B. dendrobatidis* cripples the lymphocyte mediated response [108, 109]. Apparently, soluble factors in *B. dendrobatidis* culture supernatant inhibit lymphocyte proliferation and induce apoptosis, most probably by activating apoptosis signaling pathways. These inhibitory factors have not yet been fully characterized, but seem of non-protein nature, and broadly cytotoxic. Soon after that, the same research team found that this immunosuppression is not absolute. There is proof that increased lymphocyte proliferation and abundance in the spleen can be achieved by repeated pathogen exposure and temperature-induced clearance of infection (i.e. exposure of infected amphibians for longer than 24 h to 30 °C which is the critical thermal maximum for *B. dendrobatidis*). As such, at least Oak toads (*Bufo quercicus*), Cuban treefrog (*Osteopilus septentrionalis*) and booroolong frogs (*Litoria booroolongensis*) do acquire immunity to the chytrid fungus, that overcomes *B. dendrobatidis*-induced immunosuppression [104].

Also inhibition of AMP release from dermal granular glands and selective degradation of AMP’s by fungal proteases have been suggested to contribute to reduced skin defenses against *B. dendrobatidis* (e.g. [106]). Thekkiniath et al. [110] discovered a particular subtilisin-like serine protease secreted by *B. dendrobatidis*, able to cleave certain amphibian antimicrobial peptides (AMP). Inactivation of the protective function of AMPs by pathogen-derived proteases, is a common strategy in pathogenic bacteria and fungi and contributes significantly to pathogenesis.

### Concepts of susceptibility, tolerance and resistance in chytridiomycosis

Not all amphibians respond equally to a chytrid infection and host responses can be roughly categorized as susceptible (infection resulting in disease, either followed by clinical recovery or by mortality), tolerant (persistent infection in absence of disease) or resistant (inhibition or fast clearance of infection). In chytrid-literature the term resistant (pathogen-inhibiting or pathogen-limiting) is often used for describing species that are actually tolerant (damage-limiting) and definitions may vary according to the author. More importantly, this classification is rather controversial as host susceptibility is more likely to fall along a continuum where the response of a species, population or individual host to *B. dendrobatidis* (and probably also *B. salamandrivorans*) is dictated by myriad other factors inherent to host, pathogen and environment than infection dose only. In the next paragraphs we will give some examples for each host response category. However, bearing in mind that susceptibility may vary within a species, this representation may oversimplify reality and the distinction drawn between resistant and tolerant may be disputable.

Clinical chytridiomycosis due to *B. dendrobatidis* appears to mostly occur in anurans. In truly susceptible anuran species, exposure to *B. dendrobatidis* under laboratory conditions to initial low doses of 100 zoospores can lead to 100% mortality of the experimental animals [111] and in the wild, exposure to *B. dendrobatidis* can lead to sharp declines and even extinction of a given species. In particular species that live and/or breed in permanent water or streams at higher elevations seem most susceptible. Striking examples include the neotropical toad genus *Atelopus* (harlequin frogs) which is by far the most threatened clade of amphibians with at least 30 of the 97 species presumably extinct [1] and the family of the Myobatrachidae with several taxa that are critically endangered (*Tautodactylus, Pseudophryne*) or suspected to be driven extinct by *B. dendrobatidis* (gastric brooding frogs in the genus *Rheobatrachus*) Other taxa susceptible for chytridiomycosis and sufferning from population declines/crashes can be found within, but are not limited to, the families Alytidae (e.g. *Alytes* and *Discoglossus*), Bombinatoridae (e.g. *Bombina*), Bufonidae (e.g. *Incilius periglenes*, *Epidalea calamita*, *Anaxyrus boreas*), Craugastoridae, Dendrobatidae, Hylidae (e.g. *Litoria caerulea*, *Litoria chloris*, *Litoria genimaculata*), Leiopelmatidae (e.g. *Leiopelma archeyi*), Leptodactylidae (e.g. *Leptodactylus fallax*), and Ranidae (e.g. *Lithobates chiricahuensis*, *Rana muscosa*, *Lithobates yavapaiensis*, *Lithobates tarahumarae*) [1, 32]. Far less research has been conducted on salamanders. Lethal chytridiomycosis has been reproduced experimentally only in a very small number of urodelan species, e.g. *Batrachoseps attenuatus* [112], *Bolitoglossa rufescens* [34], *Plethodon metcalfi* [113] and *Tylototriton asperrimus* [114]. Although chytridiomycosis in wild salamander populations has been described, for example in fire salamanders (*S. salamandra*) in Central Spain [36] and the endangered Sardanian newt *Euproctus platycephalus* [1, 115], the impact on population level seems by far less obvious and long-lasting than in susceptible anuran populations. One potential exception are Central American populations of plethodontid salamanders (Plethodontidae), which may also be strongly declining due to chytridiomycosis [4, 34, 64, 65]. Regarding *B. salamandrivorans*, especially non-Asian Salamandridae seem highly susceptible. So far, one known exception to this rule is the palmate newt (*Lissotriton helveticus*), which is resistant to *B. salamandrivorans* while infections are lethal to its close relative, the italian newt (*Lissotriton italicus*), with mortality occurring approximately 10 days after exposure [14]. In *B. salamandrivorans* susceptible urodelans, disease can evolve in two opposite directions: clinical recovery or mortality. Although some Asian representatives of the Salamandridae family (i.e. *Cynops pyrrhogaster*, *Cynops cyanurus*, *Paramesotriton deloustali*) are classified as “susceptible”, they are capable of limiting clinical disease. In experimentally infected individuals, infection either persists for up to at least 5 months, with infection loads up to 10^3^ zoospores and with recurring episodes of clinical disease, or is totally cleared [14].

Tolerant species are able to limit the fitness consequences of infection. Species belonging to this host response category do not succumb to *B. dendrobatidis* infection either in the wild, or under laboratory conditions, although they may be persistently infected. Therefore, they may act as carriers. While prevalence data for *B. dendendrobatidis* are abundant, information on the magnitude of the infection loads these carriers bear is scarce. From available data, the infection loads in naturally infected tolerant species such as *Xenopus laevis* (African clawed frog) [48, 116] and *Lithobates pipiens* (the Northern leopard frog) [117] are low (up to 200 zoospores). However, other tolerant species such as the widespread invasive *Lithobates catesbeianus* (bullfrog) and *Pseudacris regilla* (the pacific chorus frog) seem to be “supershedders”. In naturally infected bullfrogs, detected infection loads can run up to 10^5^ zoospores [51], while in experimentally infected *P. regilla* infection loads amount up to 10^4^ zoospores and are maintained over a 4-month period [54]. Both species may carry extremely high pathogen burdens without morbidity or mortality, which are lethal to most other species.

Truly resistant species for *B. dendrobatidis* infection are quite scarce. The European cave salamanders (*Speleomantes* spp.) seem to be a striking example of resistant species as *B. dendrobatidis* is not able to get grip on the skin, probably due to its highly efficient skin defences. In this species, experimental infections are cleared within 7–14 days and despite large sampling efforts there is zero prevalence of *B. dendrobatidis* infections in the wild despite the presence of an aggressive *B. dendrobatidis* lineage (*Bd*GPL) within its geographical range [91, 115]. Truly resistant species for *B. salamandrivorans* include all so far surveyed anurans and caecilians, and several urodelan species belonging to Asian hynobiid, ambystomatid and North-American plethodontid families [14, 37–39].

### Mediators of chytrid infection dynamics and disease outcome

As discussed in Sect. 4.3 the amphibian immune system plays a crucial role in confering resistance or tolerance to chytrid infection. However, there is still considerable variation in the response of a species, a population or an individual host to chytrid infection, that cannot be explained by variation in host defenses only. In this chapter we will highlight how slight changes in host, pathogen and environment, whether or not with direct repercussions on the immune system, may affect an individual’s or a population’s vulnerability to infection.

#### Host factors

The genetic make-up of the host may largely determine the outcome of *B. dendrobatidis* infection. In vertebrates, major histocompatibility complex (MHC) loci encode cell-surface receptors regulating the acquired immune response. In amphibians, individuals with specific MHC genotypes, seem to benefit from a higher survival rate when infected by *B. dendrobatidis* [118]. Indeed, specific conformations of the MHC molecules may promote binding to *B. dendrobatidis* antigens. Recently, Bataille et al. [119], found that at least one specific MHCII conformation (pocket 9) functions as adaptive marker for resistance to *B. dendrobatidis*. In contrast, a low genetic diversity within a species or population and consequent reduced biological fitness, may complicate the ability of a species or population to fight *B. dendrobatidis* infection [120].

Furthermore, there seems to be a direct correlation between the body temperature of frogs and their vulnerability to *B. dendrobatidis* infection. A temporary rise in body temperature above 25 °C may negatively affect *B. dendrobatidis*, since this thermal regime is suboptimal for the pathogen or, as suggested, since elevated body temperatures favour the immune response and thus promote survival. However, it is not clear whether these acute changes in body temperature are related to digestion, growth, reproduction or short exposure to a warm microhabitat [121] and coincidentally affect vulnerability for infection. Alternatively, this physiological response may be driven by pathogen recognition (so-called behavioural fever) which has been reported in a variety of invertebrates and ectothermic vertebrates [122]. There is also proof of amphibians acquiring behavioural resistance. In the study of McMahon et al. [104] frogs were more reluctant to avoid substrates infected with *B. dendrobatidis* after having been exposed to the fungus only once, followed by temperature induced clearance than naieve frogs.

Individual stress levels may also influence the outcome of infection. The glucorticoid stress hormone corticosterone increases due to physiological stress and is in charge for altering the host’s physiology and its susceptibility to a pathogen. Several studies have shown a positive correlation between increased stress levels and infection intensity [123]. However, the effect of stress seems to vary with the life history stage and species. In the study of Searle et al. [124] exogenous exposure of *Anaxyrus boreas* and *Lithobates catesbeianus* larvae and both larvae and post-metamorphs of *Rana cascadae* to corticosterone did not alter their susceptibility to infection. The interactions between environmental change, stress hormones and infectious diseases are complex, and it is not quite clear whether higher corticosterone levels due to e.g. changes in the environment, metamorphosis, breeding make individuals more susceptible to infection or if infection triggers higher corticosterone levels.

Differential susceptibility for infection is observed between larval, post-metamorphic, sub-adult and adult stages. For example, tadpoles of *Rana muscosa* can be infected by *B. dendrobatidis* without developing clinical symptoms, while in post-metamorphic animals infection induces morbidity and mortality [59]. Alternatively, *B. dendrobatidis* can negatively affect some species of amphibians at the larval stage and not others [125]. Also larvae and adult salamanders might display a differential susceptibility for infection with *B. salamandrivorans*. There are several plausible explanations for these phenomena. Just at metamorphosis the larval epidermis begins to stratify and keratinize, a process that is controlled by the thyroid hormone triidodothyronine (T3). Increased hormone levels during metamorphosis (e.g. T3, coricosteroid hormones), may trigger immune suppression and an increased susceptibility to chytrid infection [110, 126]. But also, at metamorphosis the immune system undergoes a dramatic reorganization, and in newly metamorphosed frogs immune defenses are not yet mature [126].

Finally, it is important to distinguish between individual and population level effects of chytridiomycosis. Infection may cause morbidity and mortality at individual level, ultimately leading to population decline but may just as well go unnoticed. In populations where *B. dendrobatidis* has a high impact on adult survival, increased recruitment (i.e. entry of new individuals into a population by reproduction) may be an important compensatory strategy allowing a population to recover from disease driven decline, even despite the endemic presence of *B. dendrobatidis* [127, 128]. Compensatory recruitment is only successful when *B. dendrobatidis* has a minimal impact on larvae and juveniles, combined with succesful mating by first time breeders before large increases in disease prevalence and intensity occur [128]. Moreover, populations left at low densities after disease-driven decline may recover due to altered disease dynamics; in the case of chytridiomycosis high population densities are likely to promote a rapid build-up of infection intensity and continuous reinfection of (infected) individuals [129]. More importantly, the complexity of the amphibian community may affect disease risk. This is known as the “dilution effect”: increased species richness, of both hosts and non-hosts, will reduce the impact of *B. dendrobatidis* (infection prevalence and intensity), by affecting host-pathogen contact rates and transmission [130].

#### Pathogen virulence

The virulence of *B. dendrobatidis* or its relative capacity to cause damage to its amphibian host, is isolate and genotype dependent [19, 131, 132]. So far, at least 6 major *B. dendrobatidis* lineages are being recognized, including a hypervirulent global panzootic lineage (*Bd*GPL) [19, 20, 44]. Unlike non-GPL strains, this invasive lineage is associated with massive declines and extinctions that spread in a wave-like manner once introduced into a new area and was involved in the major epizootics in the Americas, Australia, and Europe (Spain, French Pyrenees). As there are currently no isolates available from endemic northern-european amphibian populations coexisting with *B. dendrobatidis*, the genotype(s) circulating in this area is/are unknown. Yet, further collection of isolates, of both *B. dendrobatidis* and *B. salamandrivorans*, is of vital importance for gaining insights in the evolution of virulence.

Virulence of *B. dendrobatidis* increases experimentally with ambient temperatures below 25 °C. As discussed earlier, optimal growth of *B. dendrobatidis* occurs within a temperature range of 17–25 °C. Within this range, zoospores encyst and develop into zoosporangia faster than at low temperatures. However, at low temperatures, a larger number of zoospores is produced per zoosporangium, with zoospores remaining active and thus infective for a longer period [133]. As a consequence, mortality in naturally infected amphibians will be considerably higher, in the cooler months of the year in tropical and subtropical areas, while warmer temperatures at other times of the year will promote survival [134]. *B. salamandrivorans* infection and disease dynamics are likewise dictated to great extent by environmental temperature. Infection intensities of 10 000 zoospore equivalents at which mortality occurs [135] are reached twice as fast at 15 °C than at 20 °C, while at 25 °C *B. salamandrivorans* is unable to colonize skin [29].

*B. dendrobatidis* seems liable to attenuation. Strains that have been successively passaged on culture media quite rapidly display a weakened infectivity and pathogenicity when exposed to amphibians, which can however be partially restored by passage through an amphibian host [136].

#### Impact of environmental factors

Differential susceptibility to *B. dendrobatidis* observed in natural populations may be due to several abiotic, environmental factors such as season, temperature, elevation [133, 134, 137] and intensity of ultraviolet B (UV-B) radiation [138]. Especially high-elevation areas or regions with cool temperatures entail an increased risk for *B. dendrobatidis*-related declines and extinctions (e.g. [139]). Plausibly, these environmental factors may increase the vulnerability of an individual considerably, by changing the virulence of *B. dendrobatidis* and/or by altering the immune function of the host of the amphibian host.

The work of Schmeller et al. [57] illustrates well how both abiotic and biotic factors influence the probability of infection by *B. dendrobatidis* at population level. They observed a variation in prevalence of *B. dendrobatidis* among populations of common midwife toads (*Alytes obstetricans*) at different amphibian breeding sites in the French Pyrenees. At the majority of the breeding sites, prevalence of *B. dendrobatidis* infection was less than 5%, while at only few sites prevalence ran up to more than 90%. Both altitude and temperature correlated positively with prevalence and mortality but were not conclusive, as at sites with equivalent temperature regimes still substantial variation in prevalence and mortality was observed. At these particular sites, Schmeller et al. [57] found that prevalence of *B. dendrobatidis* correlates with the abundance and diversity of the aquatic microfauna in the mountain lakes. In this particular case, ciliates and rotifers were found to predate on the aquatic infectious zoospores, and lowered the environmental abundance of *B. dendrobatidis*. Also microcrustacean zooplankton e.g. water fleas (*Daphnia*) [140] graze on the spores of this chytrid fungus and are known to reduce the risk on infection in aquatic environments. Variation in the occurrence of *B. dendrobatidis* might as well coincide with variation in other biotic factors including the macroinvertebrate community structure (e.g., midge larvae, dragonflies, waterbugs and snails) [141] and the presence of green algae that interfere with *B. dendrobatidis*, either physically or by allelopathy (the release of secondary metabolites that are detrimental for *B. dendrobatidis*) [140]. Additional research in this field is necessary to fully comprehend the impact of these biotic factors.

#### Co-infection with multiple pathogens

So far, this review has focused on single pathogen interactions. In reality, amphibian hosts may be exposed to various pathogens including viruses, bacteria, non-chytrid fungi or helminths that may also cause severe pathology and mortality. In captive amphibians, chytridiomycosis due to *B. dendrobatidis* has been found concomitantly with e.g. *Ranavirus* [142], *Chlamydia pneumoniae* [143], *Aeromonas hydrophila* [144] and *Mycobaterium* spp. [144] infection. Also in the wild, co-infection with *B. dendrobatidis* and *Ranavirus* has been observed [145]. In these cases, it is difficult to determine which pathogen contributes most to morbidity and mortality or to distinguish between primary and secondary pathogen. Indeed, information on how interactions between co-occurring pathogens affect disease severity are quite scarce. A positive correlation has been found between infection by *Ranavirus* and *B. dendrobatidis* in some neotropical Hylidae, Craugastoridae and Dendrobatidae. Particularly in *Craugaster fitzingeri*, the odds of finding *Ranavirus* were significantly higher in individuals infected with *B. dendrobatidis* [145]. Also lower survival is observed in *Pseudacris regilla* larvae experimentally exposed to both the nematode *Ribeiroia* and *B. dendrobatidis* than when exposed to one of either pathogens [146]. But as discussed earlier, also abiotic environmental stressors may strongly influence disease susceptibility and might control whether interactions between pathogens occur.

In the same light, the question what would happen if both potentially lethal fungi are present in the same amphibian population is quite worry some. In Brazil, the co-occurrence of the hypervirulent *Bd*GPL and an endemic lineage (*Bd*Bz) resulted in a moderate but steady prevalence, suggestive for tempering of the most lethal lineage [46]. Besides, co-occurrence gave also rise to hybridization between both *B. dendrobatidis* lineages [20, 21]. In the Netherlands and Belgium, both *B. salamandrivorans* and *B. dendrobatidis* are present in native amphibian populations. While Dutch and Belgian amphibian communities are in coexistence with *B. dendrobatidis* [8], the newly emerged *B. salamandrivorans* has caused rapid mortality in Dutch fire salamander populations [12, 13]. The crucial question is whether co-infection by both fungi will reinforce or on the contrary temper the high lethality of *B. salamandrivorans*. In case of reinforcement, the native species richness is at stake and urges for appropriate measures to prevent and control local chytridiomycosis outbreaks.

## Conclusions

**Fig. 11:**
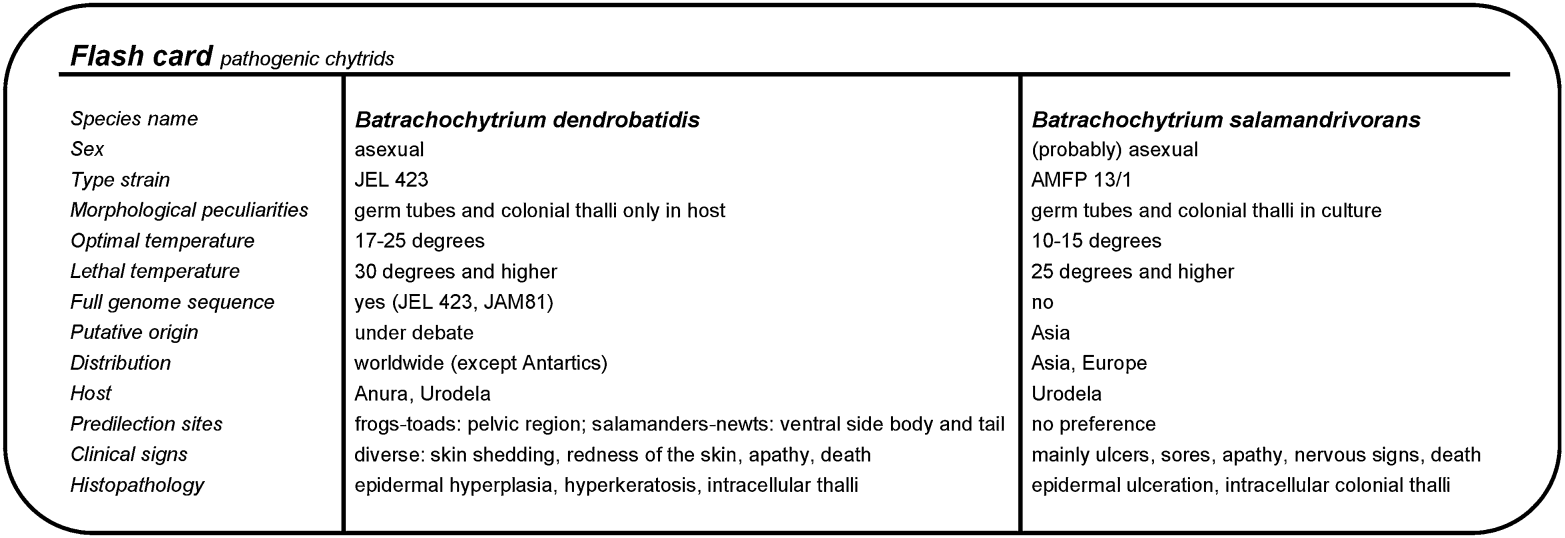
Flash card of the pathogenic chytrid fungi. Overview of the key features and most striking dissimilarities between the pathogenic chytrid fungi *B. dendrobatidis* and *B. salamandrivorans* (2015)

We have summarized all key features and most striking dissimilarities between both pathogenic chytrid fungi in a flashcard (Fig. 11). It is clear that many factors make *B. dendrobatidis* a significant concern, including its global distribution, its rapid spread, high virulence, and broad host range leading to considerable losses in amphibian biodiversity. A sobering development involves the recent emergence of *B. salamandrivorans*. Given the high susceptibility of salamanders and the current expansion of this fungus’ range in Europe, *B. salamandrivorans* poses an unprecedented threat to, at least, the western Palearctic salamander diversity. Despite the effort of various research groups to unravel the complex interactions between the chytrid pathogens, their host and the environment, there are still knowledge gaps that need to be addressed. Several big challenges for future work that came up to the fore in the review are: (1) understanding of the basic biology of these chytrid fungi, e.g. cell membrane architecture/composition might be a key factor accounting for substantial differences between both fungi and their interaction with the host; (2) elucidation of the whole infection process of both chytrid species at cellular and molecular level; (3) the further study of mechanisms driving the infection dynamics of both fungi; (4) identification of carriers and non-amphibian vectors, and other abiotic factors promoting pathogen persistence in the environment; (5) continued global mapping of distribution, prevalence and genetic diversity of *B. dendrobatidis* and in particular *B. salamandrivorans*, with special attention to areas where both pathogens might co-occur. A thorough understanding of these aspects is of vital importance, to predict future disease scenarios and for the further development of efficient mitigation and curative measures to combat chytridiomycosis.

## Additional files

10.1186/s13567-015-0266-0 Colonization of keratinous geese squamae by *B. salamandrivorans.* Experimental set-up and results from in vitro experiments examining the ability of *B. salamandrivorans* to adhere and proliferate on kerationous toe scales of waterfowl.
10.1186/s13567-015-0266-0 Experimental infection of urodelan larvae by *B. salamandrivorans.* Experimental set-up and results from in vivo infection experiments examining the ability of *B. salamandrivorans* to infect *Salamandra salamandra* and *Discoglossus scovazzi* larvae.
10.1186/s13567-015-0266-0 Chemotaxis of *B. dendrobatidis* towards amphibian skin mucus. Experimental set-up and results from in vitro experiments examining chemotaxis of *B. dendrobatidis* towards skin mucus isolated from *Xenopus laevis*.
10.1186/s13567-015-0266-0 Chemotaxis of *B. dendrobatidis* towards free integumental sugars. Experimental set-up and results from in vitro experiments examing chemotaxis of *B. dendrobatidis* towards the free integumental sugars α-L-fucose, α-D-*N*-acetylgalactosamine, β-D-*N*-acetylglucosamine, *N*-acetylneuraminic acid or sialic acid, α-D-galactose and α-d-mannose.
10.1186/s13567-015-0266-0 In vitro inhibition of *B. dendrobatidis* zoospores by amphibian skin mucus. Experimental procedures and results from in vitro assays quantifying of the zoosporicidal effect exerted by skin mucus from *Xenopus laevis*. Inhibition of *B. dendrobatidis* zoospores was quantified both visually by inverted microscopy and using EMA-qPCR.
10.1186/s13567-015-0266-0 Adhesion of *B. dendrobatidis* to *Xenopus laevis* skin. Experimental procedures of in vitro experiments examining adhesion of *B. dendrobatidis* zoospores to skin explants of *Xenopus laevis* using scanning electron microscopy.
10.1186/s13567-015-0266-0 Quantification of lysozyme in amphibian skin mucus and evaluation of its activity against *B. dendrobatidis.* Experimental procedures and results from an assay quantifying lysozyme concentrations in skin mucus from *Xenopus laevis* and in vitro assays quantifying the activity of lysozyme from chicken egg white against *B. dendrobatidis* both visually by inverted microscopy and using EMA-qPCR.
