# Supplementary material for: Amphibian chytridiomycosis: a review with focus on fungus-host interactions
Source: Vet Res. 2015 Nov 25;46:137. doi: 10.1186/s13567-015-0266-0 (PMC4660679; doi:10.1186/s13567-015-0266-0)
Supplement: Supplementary file 1 — 10.1186/s13567-015-0266-0 Colonization of keratinous geese squamae by B. salamandrivorans. Experimental set-up and results from in vitro experiments examining the ability of B. salamandrivorans to adhere and proliferate on kerationous toe scales of waterfowl. [file 13567_2015_266_MOESM1_ESM.docx]

**Additional file 1 Colonization of keratinous geese squamae by *B. salamandrivorans***

**Material and methods**

The ability of *B. salamandrivorans* (isolate AMFP 13/1) to colonize and grow on the keratinous toe scales from wild geese (*Anser anser domesticus*) was examined in vitro and performed as described in Garmyn et al. [28]. Cultivation of *B. salamandrivorans* and zoospore collection were as described in Martel et al. [13]. Toe scales were inoculated with 1 mL zoospore suspension (3 × 10^5^ zoospores/ml distilled water) and incubated at 15 °C for 24 h. Subsequently, the scales were rinsed three times in distilled water to remove non-adherent zoospores, transferred into 100 mL fresh distilled water and incubated at 15 °C for 4 weeks. Development of sporangia on the scales and release of active zoospores was evaluated daily under an inverted microscope (Olympus CKX 41, Hamburg, Germany). Finally, the scales were incubated in mPmTG-broth (0.4 g peptonized milk, 0.4 g tryptone, 2 g glucose in 1000 mL distilled water) at 15 °C, to stimulate growth and maturation of the adherent sporangia and the scales were checked daily for the presence of motile zoospores during the subsequent 7 days. The assay was performed in 3-fold.

**Results**

Immature *B. salamandrivorans* sporangia adhering and growing on the geese toe scales were observed from 5 days post inoculation onwards in 8 out of 9 wells (see Figure). Motile zoospores were absent. Beside sporangia associated with the goose toe scales no sporangia were observed elsewhere in the wells. At 14 days post inoculation motile zoospores were observed in 1 out of 9 wells. After 4 weeks incubation, zoospores were observed in 5 out of 9 wells. Addition of mPmTG-broth to the inoculated scales indeed stimulated growth and sporulation of the sporangia residing on the scales and after a 7-days incubation period abundant sporangia and zoospores were observed in 6 out of 9 wells.

**
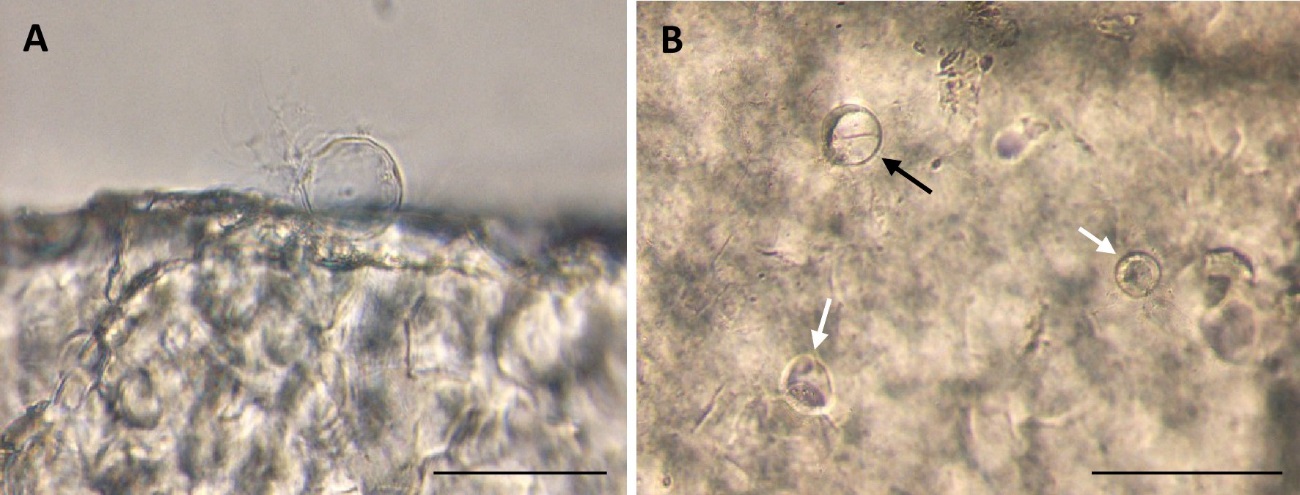
**

**Colonization of geese toe squamae by *B. salamandrivorans***. Light micrograph of geese toe squamae inoculated with *B. salamandrivorans*, showing (A) a post-discharge sporangium, scale bar: 50 µm and (B) several sporangia (black arrow: septate sporangium, white arrows: a-septate immature sporangia) present upon the surface of the keratinous squamae, scale bar: 100 µm.
