# Supplementary material for: Amphibian chytridiomycosis: a review with focus on fungus-host interactions
Source: Vet Res. 2015 Nov 25;46:137. doi: 10.1186/s13567-015-0266-0 (PMC4660679; doi:10.1186/s13567-015-0266-0)
Supplement: Supplementary file 2 — 10.1186/s13567-015-0266-0 Experimental infection of urodelan larvae by B. salamandrivorans. Experimental set-up and results from in vivo infection experiments examining the ability of B. salamandrivorans to infect Salamandra salamandra and Discoglossus scovazzi larvae. [file 13567_2015_266_MOESM2_ESM.docx]

**Additional file 2 Experimental infection of urodelan larvae by *B. salamandrivorans***

**Material and methods**

All animal experiments were approved by the Ethical Committee of the Faculty of Veterinary Medicine, Ghent University, Belgium (approval EC 2013/79) and were performed following all necessary ethical and biosecurity standards. Inoculations were carried out with *B. salamandrivorans* isolate AMFP 13/1. Cultivation and zoospore collection were as described in Martel et al. [13]. Five captive bred *Salamandra salamandra* (fire salamander) larvae and 5 *Discoglossus scovazzi* (Moroccan painted frog) larvae were housed individually and exposed to a 10 mL zoospore suspension (10^3^ zoospores/mL distilled water) for 24 h at 15 °C and were then transferred into fresh water. After 14 days the animals were sacrificed by adding an overdose of benzocaine (10 g benzocaine/100 mL EtOH) to the aquarium water. Subsequently, the mouthparts of the *D. scovazzi* larvae were excised. For each *S. salamandra* larva, skin samples were excised from the tail, hind paws, and the ventral side of the abdomen. Tissue samples were divided into two equal portions, with one portion being fixed in 10% neutral buffered formalin, processed for histology and stained with haematoxylin & eosin, and the other portion being processed for the extraction of DNA, using the DNeasy Blood & Tissue Kit (QIAGEN GmbH, Hilden, Germany) and detection of *B. salamandrivorans* DNA using quantitative real-time PCR (qPCR) techniques described by Blooi et al. [135].

**Results**

None of the anuran and urodelan larvae exposed to *B. salamandrivorans* were infected as none of the examined skin samples were positive for the presence of *B. salamandrivorans* on histology or yielded a positive amplification signal by qPCR.
