# Supplementary material for: Amphibian chytridiomycosis: a review with focus on fungus-host interactions
Source: Vet Res. 2015 Nov 25;46:137. doi: 10.1186/s13567-015-0266-0 (PMC4660679; doi:10.1186/s13567-015-0266-0)
Supplement: Supplementary file 3 — 10.1186/s13567-015-0266-0 Chemotaxis of B. dendrobatidis towards amphibian skin mucus. Experimental set-up and results from in vitro experiments examining chemotaxis of B. dendrobatidis towards skin mucus isolated from Xenopus laevis. [file 13567_2015_266_MOESM3_ESM.docx]

**Additional file 3 Chemotaxis of *B. dendrobatidis* towards amphibian skin mucus**

**Material and methods**

Chemotaxis of *B. dendrobatidis* towards mucus of *Xenopus laevis* (African clawed-frog) was determined according Moss et al. [68] and Garmyn et al. [28]. Skin mucus was isolated from 5 adult *B. dendrobatidis*-free wild type *X. laevis* (European *Xenopus* Resources Centre Portsmouth, UK). Skin mucus was harvested by gently scraping the frogs with the blunt side of a sterile scalpel blade. Mucus from all frogs was pooled and kept on ice until centrifugation for 20 min at 13 000 rpm at 4 °C to remove epidermal cell debris The supernatant was collected and stored at −70 °C until use. The total protein concentration of the mucus samples was determined using the RC-DC^TM^ protein assay (Bio-Rad Laboratories, Hercules, CA, USA) with bovine serum albumin as standard. A 5 mm cellulose acetate disc, impregnated with 100 µL mucus (0.7 mg/mL protein content) or sterile distilled water (negative controls), was placed at one side of the counting grid of a Burker counting chamber (Marienfeld, Lauda-Königshofen, Germany) and 10 µL inoculum (2 × 10^7^ zoospores/mL) was carefully added at the opposite side of the grid. Cultivation of *B. dendrobatidis* isolate IA042 and zoospores collection was as described in Martel et al. [147]. The number of zoospores in the squares adjacent to the impregnated discs were counted after 45 and 90 min incubation at 20 °C, using an inverted microscope (Olympus CKX 41, Hamburg, Germany). The assay was carried out in 4-fold. For statistical analysis, all zoospore counts were square-root transformed to ensure normality of the dataset. Normality was assessed using QQ plots and a Shapiro-Wilks test. Homogeneity of variance was evaluated using Levene’s test. One-way ANOVA with SPSS (IBM SPSS Statistics for Windows, Version 22.0. Armonk, NY, USA) (at a significance level of *p* ≤ 0.05) was conducted to determine whether crude mucus had a significant effect as attractant on *B. dendrobatidis* zoospores compared to water.

**Results**

A significant migration of *B. dendrobatidis* (*p* = 0.002) towards mucus impregnated discs could be observed, even with a fairly low protein concentration of 0.7 mg/mL mucus. No migration was observed towards discs treated with water. During their migration towards mucus about 50 to 90% of the zoospores in the grid became immobile. This was not observed in control assays with water as attractant.
