# Supplementary material for: Amphibian chytridiomycosis: a review with focus on fungus-host interactions
Source: Vet Res. 2015 Nov 25;46:137. doi: 10.1186/s13567-015-0266-0 (PMC4660679; doi:10.1186/s13567-015-0266-0)
Supplement: Supplementary file 4 — 10.1186/s13567-015-0266-0 Chemotaxis of B. dendrobatidis towards free integumental sugars. Experimental set-up and results from in vitro experiments examing chemotaxis of B. dendrobatidis towards the free integumental sugars α-L-fucose, α-D-N-acetylgalactosamine, β-D-N-acetylglucosamine, N-acetylneuraminic acid or sialic acid, α-D-galactose and α-d-mannose. [file 13567_2015_266_MOESM4_ESM.docx]

**Additional file 4 Chemotaxis of *B. dendrobatidis* towards free integumental sugars**

**Material and methods**

The sugars α-L-fucose (Sigma Aldrich, St Louis, MO, USA), α-D*-*N-acetylgalactosamine (Sigma Aldrich), β-D-N-acetylglucosamine (Sigma Aldrich), *N*-acetylneuraminic acid (Sigma Aldrich) or sialic acid, α-D-galactose (Merck Darmstadt, Germany) and α-D-mannose (Sigma Aldrich), constituting the carbohydrate portion of *Xenopus laevis* (African clawed-frog) mucins were tested as attractant for *B. dendrobatidis* (JEL 423). Cultivation of *B. dendrobatidis* and zoospore collection was as described in Martel et al. [13]. Sugars were dissolved in distilled water, filter sterilized and tested at a 0.1M concentration. Attractant concentrations of 0.2-2% wt/vol. have been shown effective for *B. dendrobatidis* [68]. N-acetylneuraminic acid solutions were adjusted to neutral pH. Hematocrit capillaries (75mm length; Hirschmann laborgeräte, Eberstadt, Germany) were filled 60 µL sugar solution, vehicle control capillaries with 60 µL sterile distilled water. To prevent leakage, the capillaries were sealed with wax plugs (Hirschmann laborgeräte, Eberstadt, Germany) at one side. Each capillary was swiped on the outside with lens paper (Kimtech Science, Kimberley Clark, Roswell, GA, USA) to remove possible attractant spillover. Capillaries were incubated in 400 µL inoculum (8-13 × 10^5^ zoospores) and placed in a holder inclined about 65 degrees upwards. The assay was incubated for 90 min at 21-23 °C, after which the capillaries were removed and swiped again at the outside to remove *B. dendrobatidis* zoospores possibly adhering on the outside. Inocula were checked for motility of the zoospores using an inverted microscope (Olympus CKX 41, Hamburg, Germany). Contents of the capillaries were collected and centrifuged for 2 min at 12 000 rpm. The supernatant was taken off as much as possible. The pellet was suspended in 100 µL Prepman Ultra Sample Preparation reagent (Applied Biosystems, Life Technologies Europe, Ghent, Belgium) and DNA was extracted according the manufacturers guidelines. DNA samples were diluted 1:10 in water. For each sample the number of *B. dendrobatidis* zoospores was quantified using a quantitative real-time PCR (qPCR) [148]. Within each assay, all sugars and negative controls were tested in triplicate. The assay was repeated in 3-fold. Statistical analyses were performed in SPSS (IBM SPSS Statistics for Windows, Version 22.0. Armonk, NY, USA). A non-parametric Kruskal-Wallis test (at a significance level of *p*≤ 0.05) was conducted to determine whether the attractants had a significant effect on zoospore movement as compared to water. A post-hoc Mann-Whitney U test (at a significance level of *p*≤ 0.05) was used to identify differences among pairs of attractants or attractant versus water. For each of the sugars tested, the Odds ratio (OR) was calculated to estimate the odds of zoospores being attracted when exposed to sugars, compared to the odds of zoospores being attracted when not exposed to sugars but to the vehicle control, water. Corresponding 95% confidence intervals and *P*-values at a significance level of *p* ≤ 0.05 were calculated.

**Results**

For each assay, the mean final number of *B. dendrobatidis* zoospores ± standard error (SEM) in each capillary containing sugar or water after a 90 min incubation period is presented in Table. There were no statistically significant differences between the experiments and therefore 3 replicate assays were evaluated as a whole. There was a significant difference in attracted zoospores between the different molecules assayed as attractantia (χ^2^(6) = 25.452, *p*< 0.001). *B. dendrobatidis* zoospores were significantly more attracted to the sugar solutions than to the vehicle control water (*p*≤ 0.008). Among the different sugars tested galactose, N-acetylglucosamine and N-acetylgalactosamine were more attractive than fucose, mannose and *N*-acetylneuraminic acid. Only compared to N-acetylneuraminic acid, the sugars galactose (*p* ≤ 0.05), N-acetylglucosamine (*p*≤ 0.05) and N-acetylgalactosamine (*p*= 0.019) were significantly more attractive.

| **Assay** | **Man** | | | **Gal** | | | **Fuc** | | | **GluNAc** | | | | **GalNAc** | | | **NeuNAc** | | | **Water** | | |
| --- | --- | --- | --- | --- | --- | --- | --- | --- | --- | --- | --- | --- | --- | --- | --- | --- | --- | --- | --- | --- | --- | --- |
|  |  |  |  |  |  |  |  |  |  |  |  |  |  | |  |  |  |  |  |  |  |  |
| **1** | 61477 | ± | 52735 | 105273 | ± | 49436 | 85558 | ± | 96573 | 72281 | ± | 65128 | 42469 | | ± | 17017 | 33465 | ± | 17303 | 11150 | ± | 754 |
|  |  |  |  |  |  |  |  |  |  |  |  |  |  | |  |  |  |  |  |  |  |  |
| **2** | 22049 | ± | 7041 | 98820 | ± | 44010 | 41368 | ± | 26671 | 65237 | ± | 74174 | 75811 | | ± | 59734 | 26338 | ± | 25258 | 10553 | ± | 334 |
|  |  |  |  |  |  |  |  |  |  |  |  |  |  | |  |  |  |  |  |  |  |  |
| **3** | 30836 | ± | 10207 | 24088 | ± | 18926 | 43050 | ± | 58815 | 34709 | ± | 16403 | 50440 | | ± | 28929 | 13784 | ± | 12231 | 5908 | ± | 279 |
|  |  |  |  |  |  |  |  |  |  |  |  |  |  | |  |  |  |  |  |  |  |  |

**Table: Chemotaxis of *B. dendrobatidis* zoospores to free integumental sugars**. Overview of the mean ± standard error (SEM) genomic equivalents of *B. dendrobatidis* detected by qPCR in each capillary after 90 min incubation. Chemotaxis was measured during 3 independent assays, carried out in triplicate. Man: mannose; Gal: galactose; Fuc: fucose; GluNAc: N-acetylglucosamine; GalNAc: N-acetylgalactosamine; NeuNAc: N-acetylneuraminic acid.
