# Supplementary material for: Amphibian chytridiomycosis: a review with focus on fungus-host interactions
Source: Vet Res. 2015 Nov 25;46:137. doi: 10.1186/s13567-015-0266-0 (PMC4660679; doi:10.1186/s13567-015-0266-0)
Supplement: Supplementary file 5 — 10.1186/s13567-015-0266-0 In vitro inhibition of B. dendrobatidis zoospores by amphibian skin mucus. Experimental procedures and results from in vitro assays quantifying of the zoosporicidal effect exerted by skin mucus from Xenopus laevis. Inhibition of B. dendrobatidis zoospores was quantified both visually by inverted microscopy and using EMA-qPCR. [file 13567_2015_266_MOESM5_ESM.docx]

**Additional file 5 In vitro inhibition of *B. dendrobatidis* zoospores by amphibian skin mucus**

**Material and methods**

A 50 µL zoospore suspension (±1.8 × 10^5^ zoospores) of *B. dendrobatidis* isolate JEL 423 was added to 200 µL of skin mucus (1.10 mg protein/mL) or 200 µL of distilled water (negative control). Zoospore collection was as described in Martel et al. [13], isolation of mucus and protein quantification were as described in Additional file 3. In each well, the number of motile and non-motile zoospores, out of a total of 100 zoospores was counted in one microscopic field of an inverted microscope (Olympus CKX 41, Hamburg, Germany), at magnification 20x. Zoospores were counted immediately after adding the zoospores to the mucus or distilled water, after 30 min. and after 1 h. The experiment was conducted in triplicate. Viability of the zoospores after 2 and 24 h incubation in mucus was assessed by using the viable/death stain ethidium monoazide (EMA; Sigma-Aldrich, Bornem, Belgium) in combination with qPCR [149]. A 25 µL zoospore suspension containing ± 2 × 10^5^ zoospores was added to 100 µL mucus, or 100 µL distilled water (negative control). Positive control samples consisted of 100 µL distilled water inoculated with 25 µL ± 2 × 10^5^ heat-killed zoospores (heated for 15 min at 85 °C). Prior to inoculation, zoospores had been washed in distilled water and centrifuged at 1500 rpm. After 2 and 24 h of incubation at 20 °C, the viability of the zoospore in the samples suspensions was verified by inverted microscopy and the number of viable zoospores was assessed using qPCR on the zoospores that were pretreated with EMA [149]. The zoosporicidal activity of skin mucus was quantified for 3 biological replicates (mucus was collected at different intervals; for each biological replicate the mucus from 5 frogs was collected and pooled), each comprising 3 technical replicates. Killing activity was expressed as log(10) viable spores added to the skin secretions – log(10) viable spores recovered after 2 and 24 h incubation. Normality of each dataset was evaluated using a QQ plot and a Shapiro Wilks test. The differences in both motility and viability of *B. dendrobatidis* zoospores when incubated water or mucus was evaluated using the non-parametric Mann Whitney U test. All statistical analyses were carried out with the SPSS software (IBM SPSS Statistics for Windows, Version 22.0. Armonk, NY, USA).

**Results**

As shown in Figure, immediately after adding the zoospores to mucus, the mean percentage of motile zoospores decreased to 30.23 ± 16.04% and differed significantly when compared to water (100 ± 19.26%; *p* ≤ 0.05), and decreased further in the course of the incubation period to a mean zoospore motility of 18.17 ± 6.63% after 30 min incubation in mucus (versus 100 ± 47.98% in water; *p* ≤ 0.05) and 7.10 ± 7.29% after one hour incubation in mucus (versus 100 ± 20.11% in water; *p* ≤ 0.05).


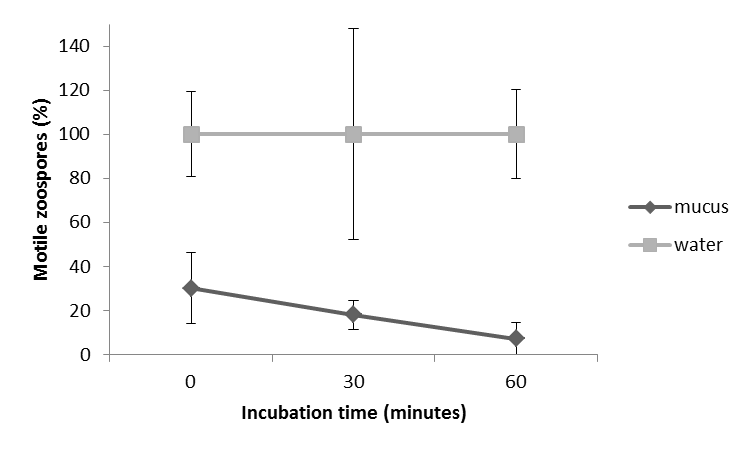


**Figure: Inhibition of *B. dendrobatidis* in skin mucus of *Xenopus laevis***. Inhibition is expressed as the percentage of motile zoospores observed in amphibian skin mucus, in function of the percentage motile zoospores observed in water. Results are presented as mean ± standard error (SEM) zoospore counts.

The killing activity of skin mucus and its components at physiological concentrations was evaluated. Skin mucus has indeed a zoosporicidal effect. However, the magnitude of this effect varied between the experiments and the mucus samples tested (Table 1). This may be due to the fact that interval between the collection of the mucus samples varied (2 days between experiment 1 and 2; 4 days between experiment 2 and 3) but also inter-individual differences may have caused variation. Also the protein concentrations of the mucus samples used varied (1.93, 1.49 and 1.50 mg protein/mL, respectively). The protein fraction in mucus is predominantly composed of mucin glycoproteins, but may also include antimicrobial peptides, hormone-like peptides, metalloendopeptidases or lysozyme [70,77]. During each experiment, the zoosporicidal effect increased during the course of the 24 h incubation period. In vitro exposure of *B. dendrobatidis* zoopores to *X. laevis* skin mucus and its components at physiological concentrations, resulted in a mean 3.60 ± 2.47-fold decrease in viable zoospores after 2 h, corresponding with a reduction up to 73% of the initial zoospore load and a 21.57 ± 10.18-fold decrease 24 h post exposure, corresponding with a reduction up to 95% in the initial zoospore load. The amount of viable zoospores in mucus compared to water was significantly lower for all experiments and all sampled time points (*p* ≤ 0.05), with the exception of experiment 1 where the amount of viable zoospores detected in mucus after 2 hours incubation did not differ significantly (*p*= 0.83) from the amount detected in water (Table 2).

| **Time point (h)** | **Viability reduction ± SEM (log)** | **Viability reduction ± SEM (factor)** | **Residual viable *Bd* ± SEM**  **(%)** |
| --- | --- | --- | --- |
| 2 | 0.46 ± 0.31 | 3.60 ± 2.47 | 43.52 ± 29.28 |
| 24 | 1.25 ± 0.33 | 21.57 ± 10.18 | 7.73 ± 8.14 |

**Table 1 Zoosporicidal activity of *Xenopus laevis* skin mucus at physiological concentrations**. Killing activity is expressed as log(10) viable spores added to the mucus– log(10) viable spores recovered after 2 and 24 h incubation. Results are presented as mean genomic equivalents of *B. dendrobatidis* (*Bd*) ± standard error (SEM) from 3 biological replicates, comprising 3 technical replicates.

|  | **Time point (h)** | **viable *Bd* in mucus** | **viable *Bd* in water** | ***P*-value** |
| --- | --- | --- | --- | --- |
| Replicate 1 (*n*=3) | 2 | 2.01 ± 0.17 | 2.83 ± 0.06 | 0.83 |
|  | 24 | 1.56 ± 0.19 | 2.51 ± 0.25 | 0.05* |
| Replicate 2 (*n*=3) | 2 | 1.98 ± 0.29 | 2.60 ± 0.16 | 0.05* |
|  | 24 | 0.89 ± 0.14 | 2.35 ± 0.04 | 0.05* |
| Replicate 3 (*n*=3) | 2 | 1.20 ± 0.58 | 1.80 ± 0.13 | 0.05* |
|  | 24 | 0.23 ± 0.13 | 1.58 ± 0.06 | 0.05* |
| **Overall mean** | 2 | 2.03 ± 0.73 | 2.41 ± 0.48 |  |
|  | 24 | 0.89 ± 0.56 | 2.15 ± 0.42 |  |

**Table 2** **Viability of *B. dendrobatidis* zoospores in *Xenopus laevis* skin mucus at physiological concentrations.** Experiment and overall mean of viable spores after 2 and 24 h incubation in skin mucus. Results are presented as mean genomic equivalents of *B. dendrobatidis* (*Bd*) ± standard error (SEM) from 3 biological replicates, comprising 3 technical replicates. (*) *P*-values indicating a significant difference between the reduction in zoospore viability in mucus compared to water.
