# Supplementary material for: Amphibian chytridiomycosis: a review with focus on fungus-host interactions
Source: Vet Res. 2015 Nov 25;46:137. doi: 10.1186/s13567-015-0266-0 (PMC4660679; doi:10.1186/s13567-015-0266-0)
Supplement: Supplementary file 6 — 10.1186/s13567-015-0266-0 Adhesion of B. dendrobatidis to Xenopus laevis skin. Experimental procedures of in vitro experiments examining adhesion of B. dendrobatidis zoospores to skin explants of Xenopus laevis using scanning electron microscopy. [file 13567_2015_266_MOESM6_ESM.docx]

**Additional file 6 Adhesion of *B. dendrobatidis* to *Xenopus laevis* skin**

**Material and methods**

All animal experiments were performed following all necessary ethical and biosecurity standards as defined by the Ethical Committee of the Faculty of Veterinary Medicine, UGent, Belgium. Adhesion of *B. dendrobatidis* zoospores to mucus covered epithelium of *Xenopus laevis* (African clawed-frog) was studied in an Ussing chamber based infection model. Isolation of full thickness epidermal explants (FTE) explants from *X. laevis* skin, in *vitro* culture conditions and infection protocols were carried out according Van Rooij et al. [24,75]. Inoculations were carried out with *B. dendrobatidis* isolate JEL 423. Explanted skin was exposed to 7 ml inoculum (2 x 10^7^ zoospores/ml distilled water) for 24 and 48 hours at 20°C. Samples were fixed and processed for scanning electron microscopy (SEM) as described in Garmyn et al*.* [28].
