# Supplementary material for: Amphibian chytridiomycosis: a review with focus on fungus-host interactions
Source: Vet Res. 2015 Nov 25;46:137. doi: 10.1186/s13567-015-0266-0 (PMC4660679; doi:10.1186/s13567-015-0266-0)
Supplement: Supplementary file 7 — 10.1186/s13567-015-0266-0 Quantification of lysozyme in amphibian skin mucus and evaluation of its activity against B. dendrobatidis. Experimental procedures and results from an assay quantifying lysozyme concentrations in skin mucus from Xenopus laevis and in vitro assays quantifying the activity of lysozyme from chicken egg white against B. dendrobatidis both visually by inverted microscopy and using EMA-qPCR. [file 13567_2015_266_MOESM7_ESM.docx]

**Additional file 7 Quantification of lysozyme in amphibian skin mucus and evaluation of its activity against *B. dendrobatidis***

**Material and methods**

Lysozyme activity in mucus samples isolated from 4 *Xenopus laevis* (African clawed frogs) individuals and with respective protein concentrations of 0.16, 0.34, 0.50 and 0.96 mg/mL (quantified as described in Additional file 3) was determined using the EnzChek®Lysozyme Assay kit (Molecular Probes, Life Technologies, Merelbeke, Belgium). For details on the experimental animals and isolation of mucus, we refer to Additional file 3. Briefly, 50 μL of each mucus sample was diluted with 50 µL reaction buffer (0.1 M sodium phosphate, 0.1 M NaCl, pH 7.5) and incubated with 50 μL fluorescein labeled *Micrococcus lysodeikticus* (50 μg/mL) for 30 min at 37 °C. The fluorescence was measured in a fluorescence microplate reader using excitation/emission wavelengths of 485/527 nm. Background fluorescence, determined for a no-enzyme control, was subtracted from each value. The lysozyme activity of the samples was calculated from a standard curve prepared with lysozyme from chicken egg white (Sigma Aldrich, Bornem, Belgium), containing a 2-fold dilution series ranging from 250 to 4 U/mL. Subsequently, the minimal inhibitory concentration (MIC) of lysozyme from chicken egg white for *B. dendrobatidis* (isolate JEL 423) was determined using a macrodilution method in 24-wellplates (Cellstar®, Greiner Bio-One, Wemmel, Belgium) as described by Martel et al. [147]. To each well, 200 μL of TGhL broth containing various concentrations of lysozyme were added to 200 μL zoospore suspension (2 × 10^5^ zoospores) to obtain as final assay concentrations of lysozyme 1, 2, 4, 8, 16, 32, 64 or 128 U/mL. Plates were sealed and incubated at 20 °C. The MIC value was determined as the lowest concentration of lysozyme at which no growth of *B. dendrobatidis* was observed. In a first experiment growth was assessed after 5 days incubation at 20 °C using an inverted microscope (Olympus CKX 41, Hamburg, Germany).

In a second experiment with the same set-up (identical lysozyme concentrations and inoculum) as the experiment described above, the inhibitory activity of 1 up to 128 U/mL lysozyme on *B. dendrobatidis* was assessed using both inverted microscopy and EMA-qPCR [149]. Inhibitory activity of the lysozyme concentration tested was expressed as log(10) viable spores added to the lysozyme solution– log(10) viable spores recovered after 24 h incubation. The assay was carried out in triplicate. For statistical analysis, normality of the data was evaluated using QQ plots and a Shapiro-Wilks test. Given non-normality of the data, differences in viability of *B. dendrobatidis* zoospores when incubated in water or the various lysozyme concentrations was evaluated using the non-parametric Mann Whitney U test. All statistical analyses were carried out with the SPSS software (IBM SPSS Statistics for Windows, Version 22.0. Armonk, NY, USA).

**Results**

In vitro activity against lyophilized *M.* lysodeikticus cell walls was observed, indicating the presence of lysozyme or lysozyme-like proteins in the tested mucus samples. Mean ± standard error (SEM) lysozyme activity in the mucus samples tested containing was estimated 17.4 ± 5.7U/mL. MIC assays using commercial lyzozyme from chicken egg white with concentration ranging from 1 to 128 U/mL, evaluated both microscopically and by EMA-qPCR did not demonstrate any fungicidal effect against *B. dendrobatidis*. The reduction in viability observed in the various lysozyme concentrations tested did not differ significantly from the reduction in viability observed in water (*p* = 0.134) Mean ± standard error (SEM) reduction in zoospore viability when exposed to lysozyme was log 0.18 ± 0.56, corresponding with a 1.58 ± 0.56 fold reduction, while in water a log 0.26 ± 0.06 reduction was observed, corresponding with a 1.84 ± 0.25 fold reduction in viability.
